# Improving access to breast cancer screening and treatment in Nigeria: The triple mobile assessment and patient navigation model (NCT05321823): A study protocol

**DOI:** 10.1371/journal.pone.0284341

**Published:** 2023-06-13

**Authors:** Adeleye Dorcas Omisore, Olalekan Olasehinde, Funmilola Olanike Wuraola, Elizabeth Jane Sutton, Varadan Sevilimedu, Oluwatosin Zainab Omoyiola, Anya Romanoff, Israel Adeyemi Owoade, Aanuoluwapo Feyisayomi Olaitan, T. Peter Kingham, Olusegun Isaac Alatise, Victoria Lee Mango

**Affiliations:** 1 Department of Radiology, Obafemi Awolowo University, Ile Ife, Osun State, Nigeria; 2 African Research Group for Oncology, Obafemi Awolowo University Teaching Hospitals Complex, Ile Ife, Osun State, Nigeria; 3 Department of Surgery, Obafemi Awolowo University, Ile Ife, Osun State, Nigeria; 4 Department of Radiology, Memorial Sloan Kettering Cancer Center, New York, New York, United States of America; 5 Department of Epidemiology and Biostatistics, Memorial Sloan Kettering Cancer Center, New York, New York, United States of America; 6 Department of Morbid Anatomy and Forensic Medicine, Obafemi Awolowo University Teaching Hospitals Complex, Ile Ife, Osun State, Nigeria; 7 Department of Surgery, Memorial Sloan Kettering Cancer Center, New York, New York, United States of America; 8 Department of Global Health and Health System Design, Icahn School of Medicine at Mount Sinai, New York, New York, United States of America; Iranian Institute for Health Sciences Research, ISLAMIC REPUBLIC OF IRAN

## Abstract

**Background:**

In Nigeria, breast cancer incidence is rising, late presentation is common, and outcomes are poor. Patient-related factors such as lack of awareness and misperceptions in addition to health system deficiencies such as lack of a clearly defined framework for breast cancer screening and referral are some of the major drivers of this poor outlook. Guidelines for breast cancer screening in high-income countries have limited applicability in low-middle-income countries, hence the need for innovative, resource-compatible strategies to combat the negative trend. This manuscript presents our study protocol which aims to evaluate the impact of a novel breast cancer early detection program developed to address delayed presentation and lack of access to diagnostic and treatment facilities in South-West Nigeria. This entails the use of mobile technology (innovative handheld iBreast Exam [iBE] device, mobile breast ultrasound, and mobile mammography) and patient navigation as interventions at the community level.

**Methods:**

The study (ClinicalTrials.gov identifier: NCT05321823) will adopt a randomized two group clinical trial design with one local government area (LGA) serving as an intervention arm and another serving as the control. Both LGAs will receive breast cancer awareness education but only one will receive the interventions. In the intervention arm, asymptomatic (40–70 years) and symptomatic (30–70 years) women will be invited for breast evaluation which will be performed by trained Community Health Nurses using Clinical Breast Exam (CBE), and iBE. Those with positive findings will proceed to imaging using mobile mammography and ultrasound brought to the LGA every month. Symptomatic women with negative findings on CBE and iBE will be scheduled for repeat clinical evaluation on a short-term basis (one month). The Radiologist will obtain core needle biopsies as indicated and transfer them for prompt pathological assessment. Women presenting to the Primary Healthcare Centers in the control LGA will be referred directly to Obafemi Awolowo University Teaching Hospitals Complex as per the current standard of care. Records of all breast cancer cases seen in the two LGAs during the study period will be obtained. The program metrics will include screening participation rate, cancer detection rate, stage at diagnosis, and timeline from detection to initiation of treatment. The stage at diagnosis and timeline from detection to treatment compared between the two LGAs will be used to assess the impact of the intervention. The study is proposed for 2 years; however, a descriptive analysis will be carried out at 1.5 years to evaluate the retention of the study participants.

**Study significance:**

It is anticipated that this study will provide vital data to support wider breast cancer screening efforts in Nigeria.

## Introduction

Breast cancer is a major public health challenge in Nigeria with an incidence of 52/100,000 [1], three-fold higher than the incidence four decades ago. Breast cancer incidence is projected to continue to increase in the coming decades. More worrisome, however, is the pattern of presentation–up to 80% of breast cancer cases in Nigeria present in advanced stages [2, 3]. This contributes significantly to high breast cancer mortality rates. This pattern of late presentation has remained unchanged over the last three to four decades [4]. Consequently, when combined with limited access to treatment overall, breast cancer survival in Nigeria is poor compared to high-income countries (HICs) [5–7]. Given the projected rise in breast cancer incidence, there may be an epidemic of advanced breast cancer cases without effective intervention in the coming years [8]. In addition to some recognized biological factors accounting for aggressiveness, it is clear that correctible epidemiological and system-related factors play major roles in late-stage presentation [9]. Both patient and system-related factors contribute significantly to delayed presentation [10]. Delayed presentation and lack of access to diagnostic and treatment facilities are recognized principal factors accounting for poor outcomes in low-middle-income countries (LMICs) [11, 12]. Patient-related factors include lack of breast cancer knowledge, fear of a cancer diagnosis and treatment, use of traditional and alternative remedies, and financial constraints. Awareness creation, community education, and advocacy to address erroneous patient beliefs must therefore form the basis of any early detection program. In addition, a lack of accessible breast cancer screening and the absence of a clearly defined clinical pathway for evaluating women with breast masses, coupled with limited access to diagnostic and treatment facilities, are key system factors requiring intervention [13].

Currently, there are no local data to support a specific breast cancer screening model in Nigeria. Developing a successful breast cancer screening program in a resource-limited setting requires substantial logistical and cultural considerations. Although mammography is recommended as the gold standard based on data from HICs, there are personnel, financial, and infrastructural challenges that limit its applicability for routine use in Nigeria [14].

This underscores the need for a cost-effective screening model tailored to the available human and infrastructural capabilities and designed to meet individual patient needs.

Various guidelines, such as the Breast Health Initiative Resource Stratified guidelines, have been proposed for context-appropriate breast cancer screening in resource-limited settings [15]. This includes the role of primary health care workers in performing CBE, creating awareness, and patient navigation for prompt treatment. Drawing from experiences of countries that have successfully demonstrated the feasibility of breast cancer screening performed by community workers using less expensive modalities, such as CBE, it is logical to build on such models [16–18].

The iBreast Exam (iBE) (UE Life Sciences, Philadelphia, PA) is a 510(k) FDA-cleared device that utilizes sensors to electronically palpate the breast when pressed against the skin. It can be used with minimal training. It does not distinguish malignant versus benign findings but identifies women who should undergo additional evaluation for a possible abnormality. This could be particularly useful in low-resource settings with limited breast imaging available [19].

A previous study in our population has shown that the combination of the iBE device with CBE has higher sensitivity for detecting suspicious breast lesions when compared to CBE alone (92%, 95% CI 77–98 vs 83%, 95% CI 67–94 respectively) [19]. This research builds on our previous results and findings of a study utilizing iBE in India [20]. This study proposes an early detection model that combines iBE with CBE to reduce the subjectivity of CBE, as the basic breast cancer screening tools for eligible women in the community and breast imaging (mobile mammography and ultrasound) for selected women based on clinical history, CBE, and iBE findings. The components of the triple mobile assessment in this proposed project are nurse breast exams (with iBE and CBE), mobile portable ultrasound, and mobile mammography.

This manuscript aims to describe the protocol for screening women between 40 and 70 years and diagnostic evaluation of women aged 30–70 years with breast symptoms in the first comprehensive community-based breast cancer program that offers screening, and diagnostic evaluation and provides a referral pathway and patient navigation for breast cancer treatment in Nigeria.

We hypothesize that this study will increase breast cancer screening participation rates, facilitate increased uptake of breast evaluation in communities with poor geographical access to standard breast imaging, reduce the incidence of advanced breast cancer cases, and shorten patients’ timelines from presentation to diagnosis and initiation of treatment.

## Materials and methods

### Aims

The goal of this study is to establish a novel community-based breast cancer program to address delayed presentation and lack of access to diagnostic and treatment facilities in South-West Nigeria. This study aims to develop a routine community-based breast cancer screening/evaluation program incorporating awareness creation, iBE device, CBE, mobile mammography, and portable ultrasound in a selected intervention LGA; to create a referral pathway from the community to the tertiary hospital for women with positive findings in the intervention LGA; and evaluate the impact of this proposed program on the cancer detection rate (CDR), stage at diagnosis and treatment timelines in the intervention LGA compared to a non-intervention control LGA.

### Study design and setting

This project will be carried out in Osun State, Nigeria. Osun State, located in southwest Nigeria, comprises thirty Local Government Areas (LGAs). The study will use a randomized two group clinical trial design with one LGA serving as the intervention arm (Ife North LGA) and another LGA serving as the control arm (Ife East LGA). The two LGAs are located within the same state and consist of a homogenous group of people with similar demographics, culture, language, belief system and occupational pattern. Both Ife North and Ife East LGAs are made up of 10 wards each with an estimated population of 153,694 and 188,087 respectively based on the 2006 National census. This was projected to be 188,000 and 231,000 respectively in the year 2020 using annual population change of 1.6% for Nigeria between 2006 and 2022 [21]. The population projection assumes the same rate of growth for all LGAs within a state. Each ward is served by a dedicated primary healthcare centre (PHC). The two LGAs are similar in that they are in the same State with similar sociocultural characteristics and are within the catchment area of the Obafemi Awolowo University Teaching Hospitals Complex (OAUTHC), which is the referral centre for the two LGAs. Both LGAs will receive awareness and education, but only the intervention LGA will receive community-based breast evaluation and navigation. However, the control LGA located at coordinates; latitudes 7° 31′ N and 7° 34′ N and longitudes 4° 30′ E and 4° 34′ E has adequate access to screening and diagnostic evaluation because it is about 4 kilometers to the OAUTHC where radiological (including ultrasound, mammography, computerized tomography, and magnetic resonance imaging services), pathological and surgical specialty services for breast cancer screening and management are available. The intervention LGA located at coordinates; latitude 6° 58′ and 7° 35′ N and longitude 4° 22′ and 4° 37′ E is about 25 kilometers form OAUTHC. The referral center (OAUTHC) is in Ife central LGA and falls within the latitude 07° 30^′^ 0.0^″^ to 07° 31^′^ 6.71^″^ and within longitude 4° 33^′^ 0.0^″^ to 3° 34′ 30.64^″^.

### Sample size calculation

#### Sample size of study participants

Three wards in Ife North (Ipetu II, Edunabon I and Moro) and two wards in Ife East (Modakeke I and Modakeke II) have been randomly selected to serve as the intervention and control arms respectively. The investigators anticipate the problem of contamination by potential study participants in the control arm who live in wards that share a boundary and are in proximity with the intervention arm. This was mitigated by excluding the wards in the control LGA located at the boundary region between the two LGAs before carrying out the random sampling to select the wards in the two LGAs. Okooko, Edunabon and Moro PHCs which serve Ipetu II (with screening population of about 2926), Edunabon I (with screening population of about 998) and Moro (with screening population of about 1876) wards respectively in Ife North LGA as well as Adeowo and Okebode PHCs which serve Modakeke I (with screening population of about 2251) and Modakeke II (with screening population of about 2249) wards respectively in Ife East LGA have been selected as the 5 PHCs where recruitment of women into the study will take place.

The 3 selected wards in Ife North LGA have an estimated total screening population (women 40–70 years) of 5,800 while the 2 selected wards in Ife East LGA have an estimated total screening population of 4,500. With an anticipated participation rate of about 70% (which is projected from the number of women willing to undergo CBE screening in a previous study in the same state [14]), we project that about 4,100 women will undergo breast evaluation in the intervention arm during the study period. The sample size of 4100 refers to the total number of patients to be accrued broadly in the intervention arm (Ife North LGA). Even though there have been three wards randomly selected in Ife North LGA and two wards randomly selected in Ife East LGA, we will assume that the rate of recruitment of patients will be the same in the randomly selected wards. In each study arm, the CDR is expected to be the same across the wards since they are expected to be similar in terms of health provider density and risk factor profile like genetic makeup, age at first-term pregnancy, parity, and family history of patients presenting for breast evaluation. Because we expect there to be no statistically significant differences in risk factors between the wards within each LGA, we do not incorporate any design effects in sample size calculation.

#### Sample size of cases

The sample size of 4100 study participants anticipated in the intervention arm (Ife North LGA) will include both asymptomatic (40–70 years) and symptomatic (30–70 years) women that will undergo screening and diagnostic evaluations respectively. In any screening exercise, there will be symptomatic cases that will use the opportunity of screening to present for evaluation. We anticipate a CDR of at least 3.5%. This expectation is supported by our previous publication of a mixed screening and diagnostic population in this geographic region demonstrating a breast CDR of 3.5% [19]. The CDR in this study will be the number of cancers detected among all study participants, including both the symptomatic and screening arms.

Our previous data demonstrate that the mean breast tumor size from an entirely symptomatic population at OAUTHC was 10.5 cm. This is much larger than findings from our recent study from the same region which included a screening population which demonstrated an average breast cancer size of 3.3 cm [19, 22]. We therefore project that cancers in the intervention community will be follow a similar pattern.

The primary outcome measure in this study is the stage at diagnosis. To achieve this, we projected the number of cases needed to detect a difference in the proportion of cases based on stage at diagnosis between the two study arms. From our preliminary data of 25 staged cancers, there were 7 (7/12, 58.3%) and 2 (2/13, 15.38%) stage IV cancers in the control and intervention arms respectively, with a 43% difference between the two groups. In order to detect a 43% difference in stage IV diagnoses between the two study arms with 80% statistical power at an alpha of 0.05, 23 cases will be needed in each arm. Our preliminary data show 13 total cancer cases detected in 791 patients and 12 total cancer cases detected in 82 patients in the intervention and control arms, respectively, in the first year. Going by this annual accrual rate, in two years, there should be 26 cancer cases out of 1582 patients in the intervention arm and 24 cancer cases out of 164 patients in the control arm. Therefore, achieving the 23 cases required for statistical significance in the stage at diagnosis in each arm at the end of the 2-year study period is possible.

#### Sample size of community health nurses

To evaluate the projected 4,100 women over a year with approximately 220 working days, we estimate that a total of 19 examinations will be performed daily across the three selected PHCs in the intervention LGA. Three CHNs per facility (nine in total) will be trained to take focused breast history, perform the examinations (iBE and CBE), triage patients for imaging, and navigate patients through the referral pathway.

### Study participants

#### Inclusion criteria

Asymptomatic women 40–70 years of age and symptomatic women 30–70 years of age with breast-related symptoms will be included in the study.

#### Exclusion criteria

Women who live outside the selected intervention and control LGAs as well as women not meeting the age criteria. Women who are too sick to present to the PHCs on their own without support will be excluded from the study.

#### Consent

Written informed consent will be obtained by the trained CHNs from all eligible study participants after reading and understanding the participant information sheet attached to the consent form (S2 File). All questions and clarifications raised by the study participants will also be addressed by the trained CHNs.

#### Activities, approach, and intervention

**Fig 1:**
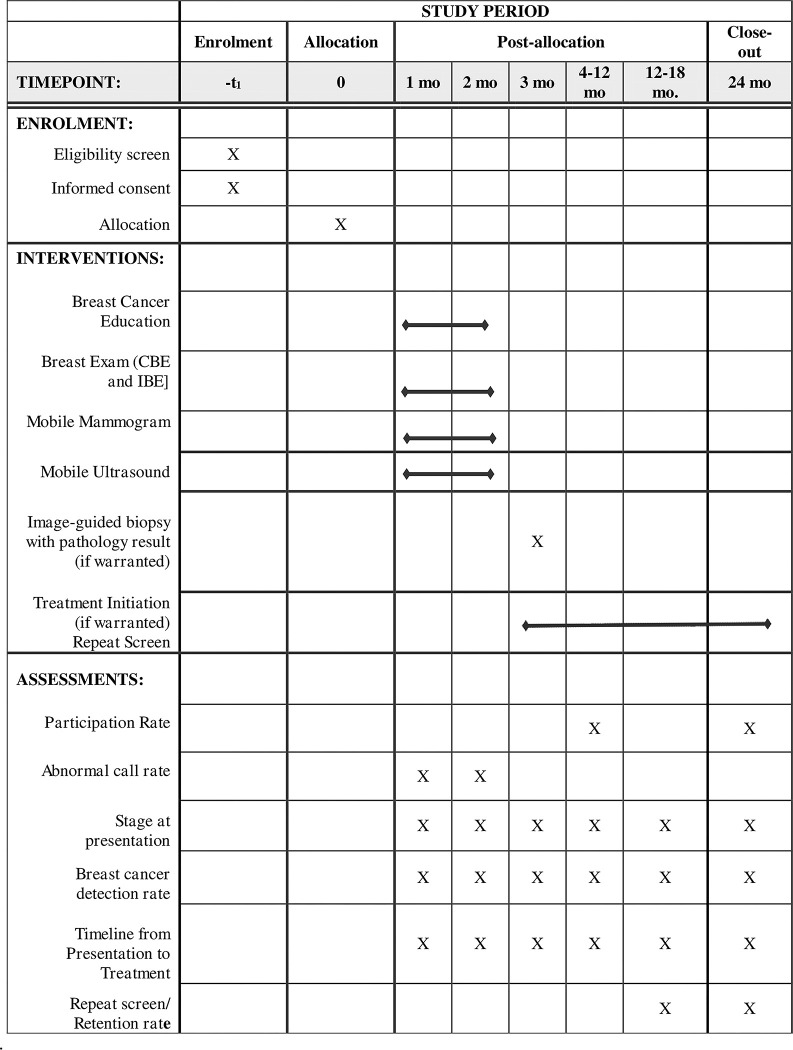
SPIRIT schedule of enrolment, interventions, and assessments.

**Fig 2:**
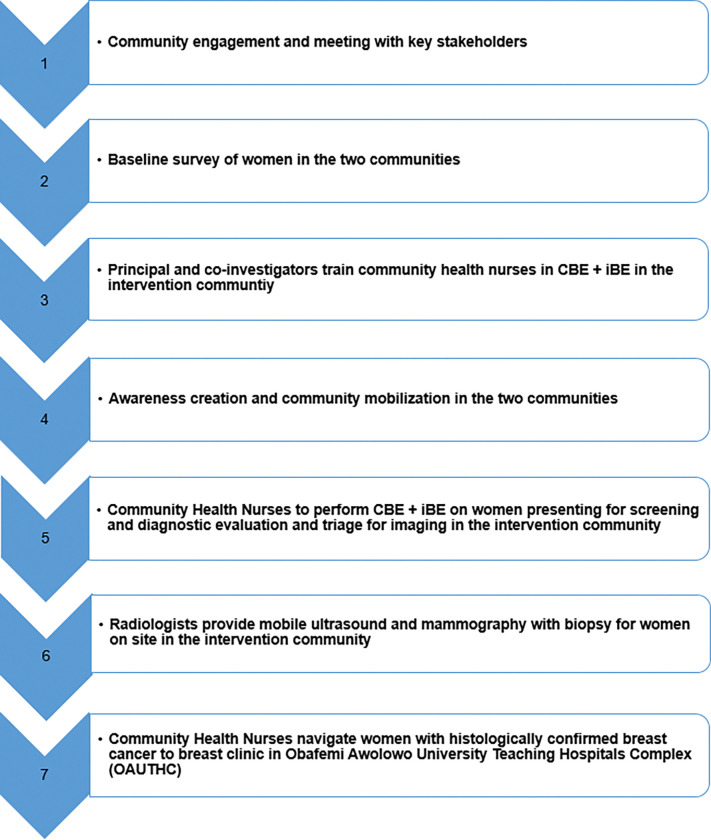
Summary of project design and methods.

The summary of the activities, approach, and intervention to be implemented is shown in Figs 1 and 2.

#### Groundwork

To guarantee broad acceptability and aid implementation, the first phase of the project entails interactions with the local district health authorities, traditional chiefs, women leaders, local health authorities, and other opinion leaders in the LGA. This research already has the support of the State Health Authorities who provided a letter of support for this project. Further consultation with the health authorities is aimed at designing an operational framework for the utilization of the primary health centres to ensure the successful integration of the screening program into the existing health system structure of the LGA. Meeting with the leaders and stakeholders is aimed at having a good understanding of the social factors that may impact the screening program, understanding the expectations of the people, and ensuring adequate community participation.

#### Baseline survey

A survey of women in the 2 LGAs (n = 163, each) will be conducted to assess baseline knowledge of breast cancer, screening practices, and perceptions about breast cancer before awareness creation and intervention. The survey instrument (S3 File) has a total of 8 sections consisting of thirty-one items. The first seven sections of the survey instrument were adopted from the validated AWACAN tool [23] while the eighth section (containing ten questions on awareness of breast cancer screening modalities and practices) was developed for this study. The English version of the survey instrument was translated into Yoruba language (the local language spoken in the selected LGAs in South-west Nigeria) and back to English language to ensure comparability. This was performed by a linguistic expert in the Department of Linguistics and African Languages of Obafemi Awolowo University (OAU), Ile Ife, Osun State, Nigeria.

The selection of women for the survey will be done using multistage sampling from the district to the street. Within each street, household sampling will be by convenience sampling of any available woman 40 years and older. The number of women sampled from each district will be proportional to the fraction of the population the district represents. Each LGA will be re-assessed with the same survey instrument (S3 File) at the end of the first year.

The CHNs will also be evaluated to assess their level of knowledge about breast cancer before intervention. This will be done both quantitatively and through qualitative interviews to understand potential cultural nuances and perceptions about breast cancer that need to be factored into the execution of the project.

#### Training staff in the intervention arm

Community Health Nurses (CHNs) in Nigeria are registered nurses and/or registered midwives with one-year post-basic training in community health nursing. They are trained to provide health education, conduct health screening, dispense medications, and administer immunization in addition to a variety of general nursing procedures like wound dressing, checking vital signs, antenatal care, and delivery, etc. Prior to this study, CHNs in the PHCs don’t have any core experience with breast cancer screening and treatment except in basic anatomy taught in nursing school and continuous nursing education seminars and training.

Three CHNs in the three selected PHCs (n = 9) will undergo a two-week training targeted at obtaining a focused breast history, performing a CBE, the use of the iBE device, counseling, and patient navigation. Training will be in the form of didactic lectures, with the use of a training manual specifically designed for this study with information derived from various sources, such as the World Health Organization training manual and materials from the American Cancer Society. Other modes of training will include role-playing, videos, and demonstrations. Training will involve education on the basics of breast cancer, such as breast cancer epidemiology, anatomy and physiology, common benign breast conditions, breast cancer risk factors, symptoms and signs, screening, diagnosis, and treatment. The health workers will be trained on how to obtain focused breast history from women presenting for evaluation. The focused history includes seven questions to assess recent breast asymmetry, breast lump, bloody nipple discharge, nipple deformity, breast skin changes, axillary swelling, and a family history of breast cancer.

Training on the performance of CBE starts with visual inspection for breast asymmetry, visible lumps, skin changes, oedema, nipple retraction, discharge, or axillary swelling while the woman is in an upright position with hands on her hips and in a supine position. Health workers will subsequently be taught how to palpate the breast using the pads of the fingers with overlapping circular movements while the woman is in an upright and then supine position with the ipsilateral arm overhead. The axillary examination will also be taught. CBE lectures will be followed by video sessions, after which the technique will be demonstrated on breast models.

Training on the use of the iBE device will be conducted following the manufacturer’s manual (UE Life Sciences Inc.) and led by one of the principal investigators of the study who has extensive experience with the device. Each health worker will be required to perform approximately 30 examinations with the iBE to achieve proficiency similar to our pilot study [19].

CBE and iBE will first be performed on models, then on volunteers. After each examination, there will be a debriefing, beginning with self-evaluation, then feedback from other trainees, and then feedback from the trainers. Training is expected to last for 2 weeks based on our previous studies which demonstrated 1–2 weeks is needed to train community breast health volunteers in these exams.

Training will also include other important components such as patient counseling, effective communication, and professional ethics, such as confidentiality and data management. After the training session, each trained health worker will give a health talk which will be evaluated by the trainers to demonstrate their ability to counsel and effectively communicate with patients.

#### Awareness creation and community mobilization in the intervention arm

Recruitment will be done via media advertisements, posters, billboards, and community awareness programs targeting all eligible women (women between 40–70 years) to participate in screening at designated PHCs.

Radio jingles in English and Yoruba, the local language in the study area, will be aired on the three major radio stations that cover the LGAs. This will be done twice daily for two weeks before the launch of the program and weekly subsequently, spanning the entire period of the study. A fifteen-minute twice-weekly local television program will also be broadcasted for two weeks before the commencement of the program. The aim of this is to sensitize people to the nature of the disease, remedy inaccurate notions about the disease, and highlight the benefits of screening. This will be followed by short weekly television advertorials updating people about the program and inviting women who are yet to be screened.

Awareness programs in the form of town hall meetings will be held quarterly in various districts across the LGAs. This will feature talks by breast cancer survivors, key opinion leaders in the LGA, and medical personnel. The awareness programs will utilize the health belief model constructs, which address the various issues associated with behaviour change.

Handbills and educational materials detailing the nature of the program, as well as educational materials about breast cancer and instructional details on breast examination will be distributed to women at strategic locations, such as markets and places of worship.

#### Screening and diagnostic evaluation schedule in the intervention arm

In the intervention arm, consecutive consenting asymptomatic (40–70 years) and symptomatic (30–70 years) women will undergo screening and diagnostic evaluations respectively. Asymptomatic women (age 40–70 years) presenting for screening at designated PHCs will be evaluated by trained CHNs. Evaluation will entail a focused breast history, CBE, and iBE. Women with positive findings on CBE or iBE will be scheduled for another visit to have breast imaging and if indicated a breast biopsy. Women with negative findings on CBE and iBE will be scheduled for a similar repeat annual evaluation. Women will, however, be told to return at any time if any abnormality is noticed before their scheduled visit. Every woman who is screened will be given a breast health card (pink card) containing her name, contact information, a summary of findings on evaluation, and the date of the next examination. A duplicate copy of the breast card will be kept at the health centre for reference and follow-up. In addition to the breast health card, a detailed record of CBE and iBE findings for each patient will be kept by the PHC Nurse.

Symptomatic women (age 30–70 years) without an obvious lesion will be evaluated like the asymptomatic population, with focused breast history, CBE, and iBE. Those with positive findings will progress to imaging while those with negative findings will be scheduled for repeat evaluation on a short-term basis (one month). If symptoms persist at the time of the next evaluation, they will progress to imaging regardless of CBE and iBE findings.

**Fig 3:**
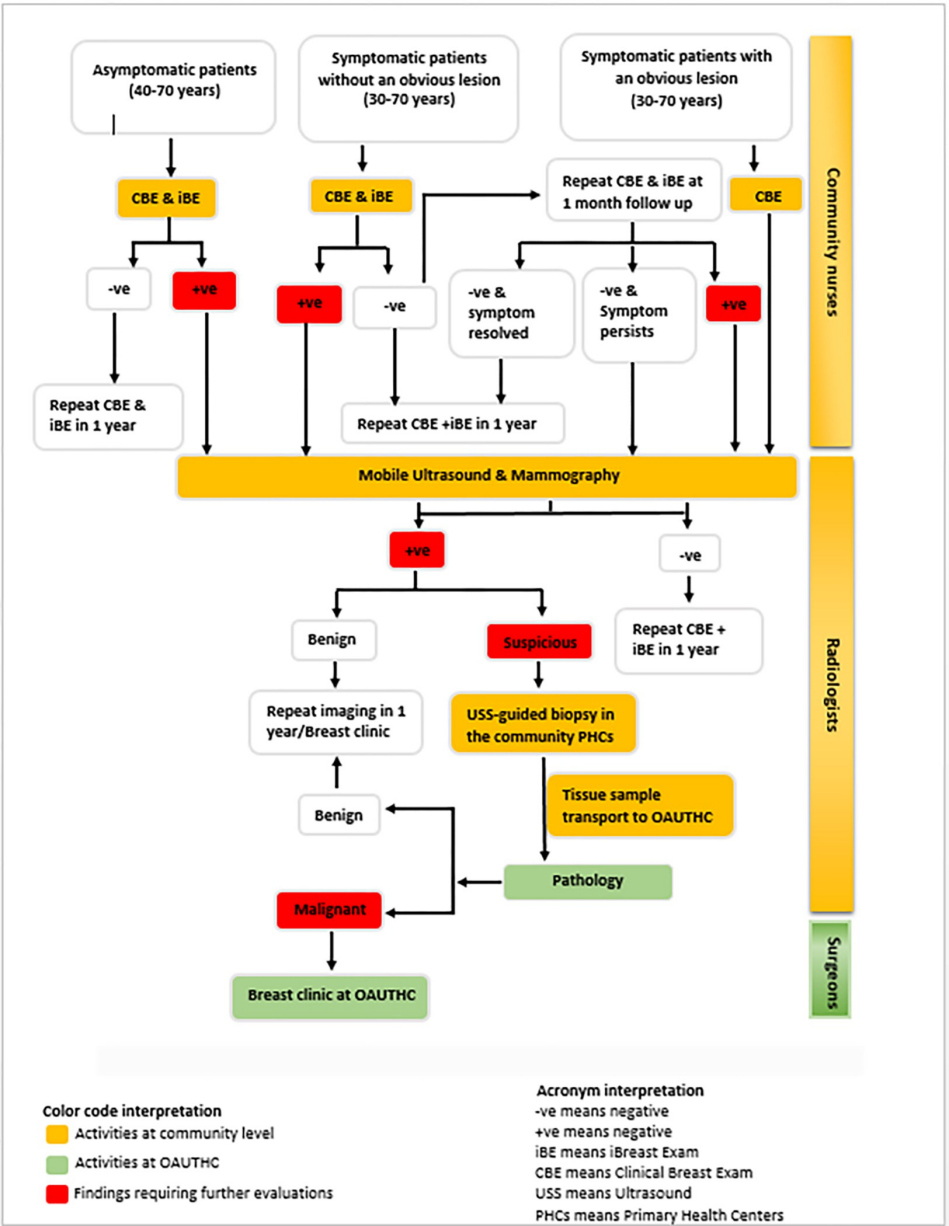
Patients’ navigation pathway from screening to treatment in the intervention community.

Symptomatic women (age 30–70 years) with obvious breast lesion(s) will undergo focused breast history and CBE examination and will be scheduled to have imaging evaluation and breast biopsy if indicated (Fig 3).

#### Evaluation of patients with positive findings in the intervention arm

Women with positive CBE or iBE findings will be scheduled for imaging, which will be conducted by the Radiologist and mammography Technologist from OAUTHC who will visit the LGA with mobile ultrasound machines and a mobile 2D digital mammography unit once a month. The 2D mobile digital mammography machine is a General Electric (GE) dedicated mammography computed radiography system secured from Lilly Women’s Health, a non-Governmental Organization in Lagos State, South-west Nigeria, which is being rented to us for the proposed two-year study period. The mobile ultrasound machines are four mHealth ultrasound tablets available in the breast unit of the Department of Radiology, OAUTHC which were utilized for a just-completed study funded under the R21 category of National Institute of Health (NIH) “Tablet-based mobile Health ultrasound for point-of-care breast cancer diagnosis in Nigeria (1R21CA239784-01)”.

Patients from all the PHCs will be seen at a central location in the LGA. Patients with suspicious findings on imaging will undergo an ultrasound-guided core needle breast biopsy on the same day. Biopsy specimens will be fixed in 10% neutral buffered formalin immediately and transported to OAUTHC the same day for processing and pathology interpretation.

### Patient navigation and follow-up in the intervention arm

Patients with histologically confirmed breast cancer will be scheduled to visit the next available breast clinic at OAUTHC. Navigation to the breast clinic will be facilitated by the CHNs after the Radiologists have communicated the biopsy results to both the patient and the nurse (Fig 3). The CHNs will also check in with the patients regularly on weekly basis through phone calls and/or home visits to ensure they comply with their appointments and follow treatment recommendations. Each patient will have a Case Report Form (CRF) (S4 File) which contains the socio-demographic and clinical data; CBE, iBE, and imaging (ultrasound and mammography) findings; pathology diagnosis (histopathology and immunohistochemistry) and treatment data. The socio-demographic and clinical data, CBE, and iBE sections of the CRF will be filled out for the study participants by the trained CHNs while the imaging, pathology, and treatment sections of the CRF will be filled out by the research study assistants (RSAs). The filled CRFs at the three PHCs will be collated and transferred to OAUTHC by the RSAs who visit the PHCs on weekly basis to monitor recruitment activities and collect paper data records for upload to REDCap in OAUTHC.

#### Control arm

The control LGA is about 4 kilometers from OAUTHC which is the referral tertiary hospital in this study. There are radiological (including ultrasound, mammography, computerized tomography, and magnetic resonance imaging services), pathological and surgical specialty services for breast cancer within OAUTHC which is also the primary institution of the study’s principal and co-investigators in Nigeria. In the control LGA, awareness creation will follow a similar procedure as outlined above for the intervention LGA. No other intervention will take place in the control arm. Women will not be invited to participate in a screening program and there is no community-based imaging, biopsy, or navigation. Women are expected to present to the PHCs if they have breast complaints which is a routine practice. Management of patients with breast-related complaints will be based on the current standard of care which is a review by the CHNs who refer or clinically manage otherwise based on his/her assessment. Twice a month, research staff will visit the selected PHCs in the control LGA to obtain data on the number and records of breast-related cases seen at the PHCs and those referred to OAUTHC. Patients referred to OAUTHC for suspicion of breast cancer will be tracked to determine the stage at diagnosis and the timelines from presentation at the PHC to eventual treatment at OAUTHC. In addition, patients presenting directly to OAUTHC from the LGA based on self-referral will also be identified and their data obtained.

Although the control arm participants do not have intervention provided in their LGA, they however get same workup, management and are navigated along the same pathway as the intervention arm participants once they present in OAUTHC for screening and diagnostic evaluation.

#### Program evaluation and outcome measures

The study timeframe is 2 years; however, a descriptive analysis will be carried out at 1.5 years to evaluate for the retention of study participants. A review will also be done at the end of year one to evaluate the progress of the research. Breast screening practices of the entire LGA will be re-assessed at the end of the first year in both the intervention and control LGAs. This will be compared with the baseline data already obtained before the commencement of the study to determine the impact of the intervention on breast cancer awareness and screening practices.

**Fig 4:**
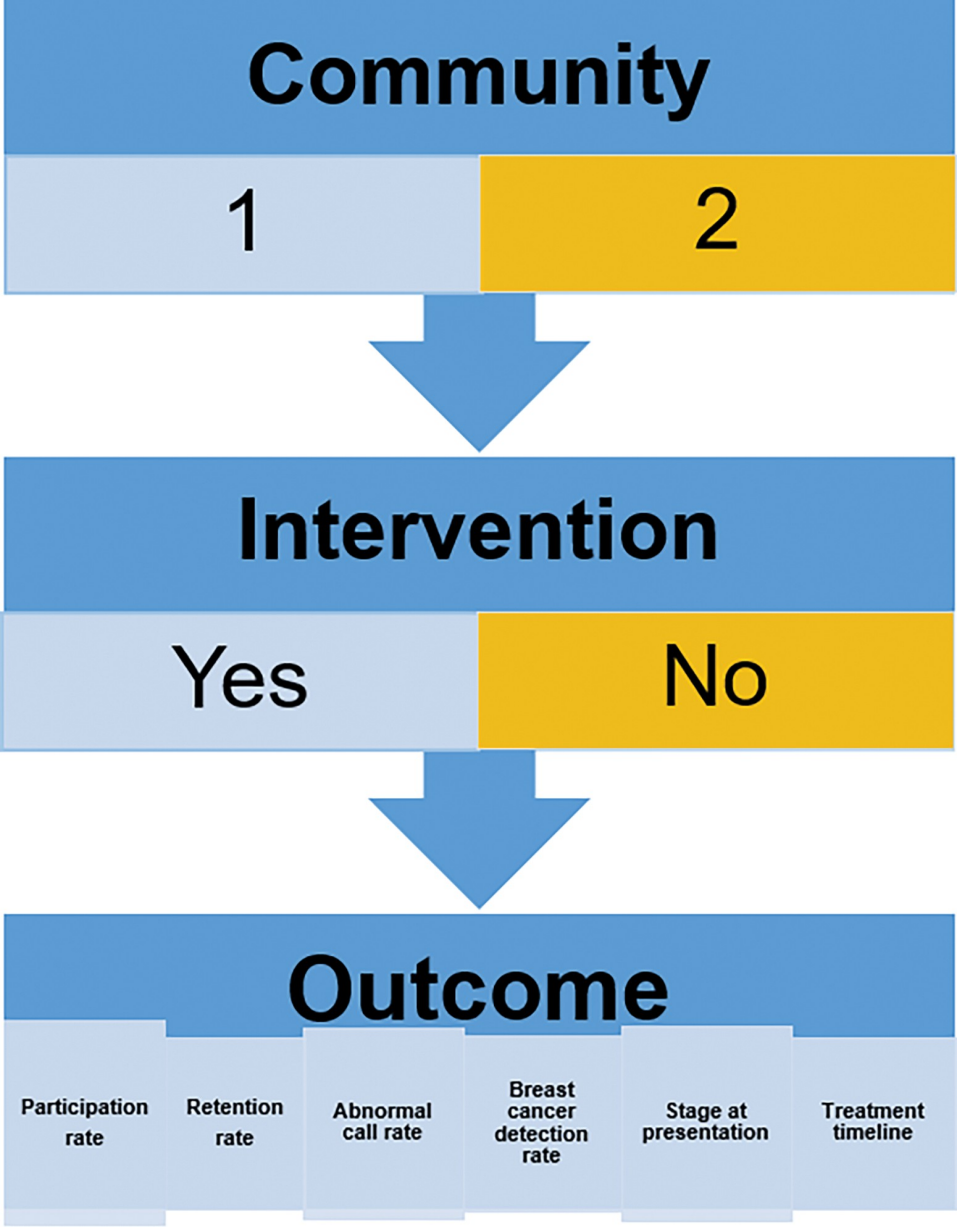
Outcome measures in the control and intervention communities.

A full evaluation will be done at the end of 2 years. The following outcomes (Fig 4) have been defined a priori and will be used as metrics to assess the impact of the program.

Primary outcome measures.

1. Stage at presentation: This will be determined using both clinical and radiological assessment to determine the TNM stage among those with histopathologically confirmed breast cancer. It is expected that the number of patients with late-stage disease may be high at the outset, but this is expected to reduce as screening progresses in the intervention arm. Therefore, besides the absolute number of cases per stage, the pattern of presentation over time will also be assessed to determine if there is a decline in the number of late-stage cases between the prevalence and incidence rounds of screening. Cases of breast cancer from the intervention and control arms will be tracked both at the PHCs and OAUTHC. The stage and pattern of presentation of cases from the two arms will be compared.

Secondary outcome measures.

1. Participation rate: This will be determined by calculating the percentage of women screened/evaluated of the total number of eligible women based on census data. A participation rate of 70% will be considered satisfactory in the intervention arm. The number of screened/evaluated women in the intervention and control arms will also be compared.
2. Cancer detection rate (CDR): This refers to the number of histopathologically diagnosed cases of breast cancer per 1,000 evaluated populations in both screening and diagnostic groups. This will be compared between the control and intervention arms.
3. Timeline from presentation to treatment: The time interval between presentation for breast evaluation and treatment in OAUTHC of the cases in the two arms will be compared.
4. Retention rate: The retention rate refers to the number of women who return for repeat annual screening of the total initial number of women initially screened in the first year. A repeat screening within a year and a half will be considered acceptable for this two-year study. To ensure all accrued participants get their second screen, we will follow them up beyond the initial study period as needed.
5. Abnormal call rate: This is defined as the number of women with abnormalities detected on iBE and/or CBE requiring further evaluation either by imaging with or without biopsy out of the total number of asymptomatic and symptomatic without an obvious lesion participating in the study. There are 2 groups of participants (diagnostic and screening) in each of the 2 arms (intervention and control). The screening group constitutes the asymptomatic participants while the diagnostic group constitutes the symptomatic participants. The interventions in this study include enhanced CBE (CBE + iBE), community-based diagnostic evaluation (with portable ultrasound and mobile mammography), and biopsy (if warranted). To evaluate which of CBE and enhanced CBE was of greatest value, another layer of analysis will be provided to address this question by comparing the concordance of the two with imaging which is the gold standard. This study also provides an additional opportunity to evaluate the iBE device.

A descriptive analysis of the data will be done at 1.5 years for the retention rate and abnormal call rate to inform our decision for future investigations. The rate of late-stage diagnosis will be estimated on a yearly basis. However, no stopping rules will be employed. This estimation will purely serve the purpose of detecting any difference in the annual trends of the stage of diagnosis over time between the intervention and control arms.

The initial findings for the subset of women who undergo a second round of screening will be examined to identify the stage of presentation in this group of women if diagnosed during the second round. Given in our setting ~80% of patients with breast cancer present in stages 3 and 4, we expect to see a trend toward earlier stage of presentation (stages 0, 1 and 2) in women undergoing repeat breast evaluation.

The 2-year study period is enough to generate adequate data to measure the participation rate and difference in stage at diagnosis between the study arms, as elaborated in the sample size calculation of the cancer cases. In addition, this study will provide a groundwork for exploring the difference in CDR between the 2 modalities (CBE and enhanced CBE) and the time to treatment, generating data to guide future studies in our population.

### Data collection and management

By local research regulations, anonymized data obtained by the CHNs will be initially recorded on paper forms and stored in a secure, locked area to ensure data security and patient privacy. A unique identifier will be assigned to each patient record. These data will be transferred and stored in Research Electronic Data Capture (REDCap) database by trained research assistants who will collect the paper data record from the various centres. The RSAs will be provided with REDCap project server login details, allowing them to securely submit data to the REDCap system. The REDCap server is managed by Obafemi Awolowo University, Ile Ife, Nigeria. All data will be collated from the centres into a central pool. Data on the total number screened at each centre and the number of those with positive findings on CBE/IBE, imaging, and biopsy will be collated every two weeks. Those referred to OAUTHC will be tracked to the final point of care to determine the stage at diagnosis and to obtain information regarding treatment. The principal investigator and research collaborators only will have access to the final study dataset.

### Statistical analyses

All data entered into the REDCap database will be exported as a dataset into R 4.2. Sociodemographic characteristics of the participants will be assessed using medians and interquartile ranges (IQR) for continuous variables and counts and percentages for categorical variables. Participation rate, retention rate, abnormal call rate, rate of late-stage diagnosis, and CDR will be reported as proportions with 95% exact confidence intervals. As for late-stage diagnosis, stage 4 cancers will be considered late-stage. The above-mentioned metrics will be compared between the intervention and control arms using Chi-square tests or Fisher’s exact test. The time intervals from presentation to treatment will be reported using medians and IQR and will be compared between the intervention and control arms using the Wilcoxon rank-sum test. The rate of late-stage diagnosis will be estimated and compared between the intervention and control arms on a yearly basis, starting from baseline, to assess if the intervention arm shows betterment in this rate consistently. Bonferroni correction for type I error will be implemented (α* = 0.0167) for this comparison. A logistic regression analysis which accounts for the ward (random effect) in which the patient was examined will also be performed with late-stage diagnosis as the dependent variable and time of screening, the interaction between the allocated groups (intervention and control arms), and time of screening as the independent variables. For all statistical comparisons (other than the one mentioned immediately above), the type I error rate (α) will be set to 0.05. All statistical analyses will be conducted using R 4.2 software [24]. Sample size calculations were done using the clinfun package of the R 4.2 software [25].

We do not anticipate any missing data due to loss of follow-up because the research team will make every effort to retain recruited participants. However, if missing data issues do arise, we will implement multiple imputation techniques, which impute missing data under the missing at random (MAR) assumption. Sensitivity analysis of the findings after multiple imputations will thereby be performed and these findings will be compared to those obtained from complete case analyses.

### Protocol and data monitoring team

The principal and co-investigators are members and clinical researchers of the African Research Group for Oncology (ARGO) which currently includes OAUTHC as the major Nigerian hospital and Memorial Sloan Kettering Cancer Centre (MSKCC) as the major United States centre. ARGO is a recognized National Cancer Institute (NCI) consortium to generate data to inform regional evidence-based management recommendations, investigate prevention and early detection strategies, increase access to cancer care, and improve cancer-care training in rural and underserved communities. The research activities of MSKCC in ARGO are coordinated through the Global Cancer Disparities Initiative (GCDI).

A protocol monitoring team (PMT) will be assigned to this study. Members of the PMT will include the GCDI clinical research manager, research project manager and clinical research coordinators, a senior breast radiologist and assistant director (VLM) of GCDI at MSKCC, and the ARGO program manager (IAO) at OAUTHC. The responsibilities of the PMT will include project compliance, data collection, abstraction and entry, data reporting, regulatory monitoring, problem resolution and prioritization, and coordinating the activities of the protocol study team. In addition to data monitoring, the activities of the study will also be monitored for safety and quality by the PMT. As this study involves a clinical trial on the FDA-approved iBE device, an independent data monitoring committee will not be formally constituted.

Assessment data will be managed through REDCap, a data management software system supported by the REDCap administrative team at OAU. Members of the OAU administrative team supporting the REDCap software will have access to the data for the purpose of ensuring the proper functioning of the database and the overall software system. REDCap is a tool for the creation of customized, secure data management systems including web-based data entry forms, reporting tools, and a full array of security features including user and group-based privileges with a full audit trail of data manipulation and export procedures. REDCap is maintained on locally-owned servers that are kept in a locked server room with appropriate environmental modifications (e.g., special air conditioning), supported by an uninterrupted power supply, and backed up nightly with some backup tapes stored off-site. All connections to REDCap utilize encrypted (SSL-based) connections. REDCap will only be used for the housing of survey and patient-level data. The use of REDCap has been approved by the OAU webmaster.

Source documentation will be available to support the computerized patient record. The principal investigator will maintain ultimate responsibility for the clinical trial.

### Quality assurance

Weekly registration reports will be generated to monitor accruals and the completeness of the registration data by the PMT. Routine data quality reports will be generated to assess missing data and inconsistencies. Accrual rates and extent and accuracy of evaluations and follow-up will be monitored periodically throughout the study period and potential problems will be brought to the attention of the protocol study team for discussion and action. Random-sample data quality and protocol compliance audits will be conducted by the PMT, a minimum of two times per year, more frequently if indicated.

### Ethical consideration and approvals

Ethical approval (ERC/2020/10/11) has been obtained from the Ethics and Research Committee (ERC) of Obafemi Awolowo University Teaching Hospitals Complex (OAUTHC). The study protocol has been approved by the primary healthcare board of Osun State in Nigeria August 19, 2020 (protocol version number ERC/2020/10/11). Any important protocol modifications will be communicated to the relevant parties via monthly study calls or via email/phone if sooner notification is warranted. The study is registered at clinicaltrials.org (NCT05321823). This study was retrospectively registered before submission to the journal but after the enrolment of participants was started because of unanticipated administrative delays. All research for all enrolled participants is conducted under full ethical and administrative institutional approvals. The authors confirm that all ongoing and related trials for this intervention are registered.

### Project status and timeline

The project is in 4 phases. The first phase (preliminaries) involves consultations with Government Health authorities, traditional rulers, chiefs, market women, and key opinion leaders and the selection of screening centres. The second phase (mobilization of resources, personnel, and participants) involves procurement of materials, training of personnel, awareness creation, and community mobilization. Implementation is the third phase which involves baseline assessment, training of CHN, recruitment of study participants, data collection, and follow-up. The fourth phase (completion phase) involves the completion of follow-up and data analysis, manuscript writing, and dissemination. The project is currently in the third phase.

## Discussion

This project is the first to assess the feasibility of a broadly applicable community-based breast cancer detection program in Nigeria. The program itself is unique in that it combines the use of low-cost mobile technology and mobile imaging for routine breast screening and diagnostic breast evaluation at the community level, thereby addressing the substantial challenges of cost and geographic access to breast cancer screening and treatment. Other conceptual innovations particular to this study include its aim to promote breast cancer screening as a routine practice rather than a one-time outreach activity, which can be a flaw of intervention programs. In addition, the concept of a vertical program leveraging existing health infrastructure by incorporation into an already existing health system is novel. This is a cost-effective model with applicability to many settings where funding is a challenge. Also incorporated into this study is the concept of patient navigation to the point of care for treatment and follow-up for those who require further care after screening. This study, therefore, provides a complete pathway–from detection to treatment–that maximizes the benefits of screening and early detection. The comprehensive design of this study, coupled with the simple but rigorous methodological approach, can generate a framework to improve breast cancer screening on a national scale.

The simple methodology deployed in this study makes it easily reproducible in other parts of the country, and the utilization of already existing health facilities and personnel (vertical approach) makes it easy to adapt on a large scale without significant financial and personnel requirements. The results of this study can also be utilized in the implementation of a similar screening program for other cancers of high public health interest in LMICs, such as prostate and cervical cancer.

To ease the implementation and integration of the results of this project into practice, relevant agencies, such as the Local Government Authority and Non-governmental Organizations are involved in the design of the project right from inception. The study team is committed to sharing research results as rapidly as possible among the investigators, the cancer research community, and relevant stakeholders. The results of our investigation will be disseminated to the scientific community via publication in international peer-reviewed journals. Our timeline and multi-pronged approach to breast cancer screening are favourable for generating multiple publications, including initial results, follow-up impact on screening, and long-term implications. Findings from this project will also be disseminated to all stakeholders in Osun State and Nigeria as a whole. Our results will be shared with the State Commissioner of Health with the intent of disseminating them for wider implementation across the State. Our findings will also be submitted to the Nigerian National Cancer Control Committee for consideration in the National Cancer Control Plan. The African Organization for Research and Training in Cancer (AORTIC) is a veritable platform for disseminating results of research projects relevant to LMICs, it is our goal to present our findings at the biennial AORTIC conference. Our findings will also be submitted for presentation at other local and international conferences, such as the West African College of Surgeons Conference, the Conference of the Breast Imaging Society of Nigeria, and the American Society of Clinical Oncology. This project will utilize the existing ARGO platform (which currently includes twenty-seven Nigerian and two United States hospitals) for the dissemination and implementation of the results of this project.

Feasibility and sustainability were central in the discussion during the design of this study. The investigators anticipate logistic constraints with the recruitment of the expected number of study participants by the recruiters who are CHNs in this study. To avoid overburdening the recruiting CHNs who perform many roles and responsibilities in the PHCs, the investigators have an understanding with the officer-in-charge (OIC) to exempt the recruiting CHNs from other responsibilities in the PHCs during the study period, which is a pragmatic way of ensuring efficient performance by the CHNs. The model proposed in this study is sustainable because of the vertical approach employed. Utilization of existing health facilities and personnel do not require significant financial and human resources commitment from the government. The government can then leverage its existing funding streams to provide the intervention package (both screening and diagnostic equipment) at a subsidized rate that will be affordable to the people.

Initially, recruitment for this study was slower than expected due to COVID-19 disruptions; however, there has been a progressive surge in the number of cases over time. This research may be challenged by the problem of contamination in the intervention and control arms. Patients particularly the symptomatic women who live outside the two selected LGAs may cross from their LGA and present in the study sites when they get informed of this research by people around them or through the ongoing radio adverts. This will be mitigated by carefully matching the residential addresses against the LGAs. This will be done by the RSAs immediately after the patients present in OAUTHC during navigation. To avoid contamination by women earlier confirmed to have breast cancer in OAUTHC, the RSAs will carefully probe into previous clinic visits by the patients in OAUTHC.

It is anticipated that this study will provide vital data to support wider breast cancer screening efforts in Nigeria through a partnership with the Ministry of Health. The results of this study could provide a breast cancer screening model for other resource-limited countries with similar population dynamics.

## Supporting information

S1 File SPIRIT checklist. (PDF)

S2 File Model consent form. (PDF)

S3 File Survey instrument. (PDF)

S4 File Case report form. (PDF)

S5 File IRB-approved study protocol. (PDF)
