# Supplementary material for: Improving access to breast cancer screening and treatment in Nigeria: The triple mobile assessment and patient navigation model (NCT05321823): A study protocol
Source: PLoS One. 2023 Jun 13;18(6):e0284341. doi: 10.1371/journal.pone.0284341 (PMC10263304; doi:10.1371/journal.pone.0284341)
Supplement: S2 File — (PDF) [file pone.0284341.s002.pdf]

PROTOCOL ELIGIBILITY CHECKLIST

Title: Improving access to breast cancer screening and treatment in Nigeria: the triple mobile assessment and patient navigation model (ERC 2020/10/11)
Pfizer tracking number: #60603257
Prinicipal investigator: Dr Adeleye D Omisore

Participant Name: (Last and First)	
Participant Research Number (REDCap Number):	Study Number:
Participant's Date of Birth (d/m/y):_______/_______/_______	Date of Consent (d/m/y):<br>_______/_______/_______
Participant's Ethnicity:<br>☐ Yoruba ☐ Hausa<br>☐ Igbo ☐ Other	Participant's Sex:<br>☐ Female
Consenting Professional/Managing Consultant: (Print Last and First)	
Research Assistant Name: (Print Last and First):	
Consent Location:<br>☐ Ife East LGA ☐ Ife North LGA	

ELIGIBILITY CRITERIA (Inclusion/exclusion criteria must be clearly defined)

Tick Response as appropriate	Yes	No
Is the participant 30years and above and symptomatic ± obvious breast lesion?<br>OR		
Is the participant between age 40 to 70 years and asymptomatic?		
Is the participant eligible for the study?		
If NO: Was eligibility override approved by the IRB? (YES/NO- If NO, the participant is ineligible)		

RESEARCH TEAM (Physician, Consenting professional or Research staff)

**Reviewed and Approved by**: (Print First and Last Name)	**Title**:
**Reviewed and Approved by**: (Signature)	**Date**: _______/_______/_______
**By signing above I attest that I have reviewed and confirmed the eligibility status of the participant.**	

PARTICIPANT INFORMATION SHEET

TITLE OF THE STUDY: Improving access to breast cancer screening and treatment in Nigeria: the triple mobile assessment and patient navigation model

Principal Investigator: Dr Adeleye Omisore
Telephone No: +2348031538004
E-mail: leyeomisore@oauife.edu.ng
Institution/Department: OAUTHC/Department of Radiology

Co – Investigators: Drs Olalekan Olasehinde and Funmilola Wuraola

i. Some general things to know about the study: The goal of this study is to establish a breast screening program for women aged 30 – 70 years in the community. We will train the Community Health Nurses in the Primary Health centers in your community on how to perform breast examination with their hands and with the use of a handheld device. The trained nurses and specialists in breast cancer diagnosis and care from Obafemi Awolowo University Teaching Hospital (OAUTHC) Ile Ife will be providing breast cancer screening and diagnosis to you in your community and guide your referral to OAUTHC for treatment if cancer is detected in your breast.

ii. What is the purpose of this study: To establish a community-based breast cancer screening program, designed to overcome the problem of poor geographic access to breast cancer screening and late presentation of women with breast lumps to the teaching hospital for treatment.

iii. Procedures: The trained Community Health Nurses will perform breast examinations on you whenever you present to them for breast cancer screening or with complaints in your breast after which you will be scheduled for an appointment with the Radiologist if the Nurses find anything abnormal on the breast examination. The Radiologist will be doing breast ultrasound and X-ray (mammography) for you within your community and may take out the abnormality they find in your breast if need be for testing in the lab to confirm cancer. If your breast abnormality is confirmed to be cancer in the lab, the trained nurses and the Radiologist will guide your referral and direct you to the breast clinic in OAUTHC to get appropriate treatment. If the breast abnormality found by the Radiologist is not cancer, you will be evaluated by the breast Surgeons for appropriate management within your community. If no abnormality is found in your breast by the Community Health Nurses and/or Radiologist, you will be rescheduled for a repeat breast screening in 1 year.

iv. Benefits: You will undergo a free breast cancer awareness education, breast screening and evaluation with mobile ultrasound and mammography, an ultrasound-guided biopsy if indicated and get free treatment support in OAUTHC if cancer is detected in your breast.

v. Costs of Participation: You will not pay for participating in the community-based breast health program.

vi. Risks: You may feel slight pain and discomfort at the time of breast compression during mammography and at the time of injection of local anaesthetic into the biopsy area during an

ultrasound-guided breast biopsy. There may be hematoma collection or bruising of the skin after the ultrasound-guided breast biopsy procedure.

vii. Compensation: You will not be compensated for this study other than having your breast screening, diagnosis and treatment performed for free.

viii. Confidentiality: Your name will not be used in the data. Your pictures may be used only upon permission

ix. Respondents' Rights: Your rights will be protected and you have the right opt-out of the study at any time.

x. Conflict of Interest: There is no conflict of interest.

xi. For the Records: The study will be carried out at no additional cost to you.

PARTICIPANT'S AGREEMENT/CONSENT FORM:

I have read the information provided in the Subject Information Sheet, or it has been read to me. I have had the opportunity to ask questions about the research and all questions I have asked have been answered to my satisfaction. I consent voluntarily to participate in this study and I understand that **I will undertake a breast cancer screening and evaluation program**. I understand that I have the right to withdraw from the study at any time.

Yes ☐ **No** ☐

____

Signature/Thumbprint of Research Respondent. Date:

____

If participants cannot read: Signature of Mother or Legal Guardian. Date:

____

Signature/tthumbprint of Person Obtaining Consent. Date:

____

Name of witness Signature
Date:

____

Printed Name of Person Obtaining Consent.
Date:
