# Supplementary material for: Improving access to breast cancer screening and treatment in Nigeria: The triple mobile assessment and patient navigation model (NCT05321823): A study protocol
Source: PLoS One. 2023 Jun 13;18(6):e0284341. doi: 10.1371/journal.pone.0284341 (PMC10263304; doi:10.1371/journal.pone.0284341)
Supplement: S3 File — (PDF) [file pone.0284341.s003.pdf]

Improving Access to Breast Cancer Screening and Treatment in Nigeria: The Triple Mobile Assessment and Patient Navigation Model

Questionnaire for Pre- and Post-Intervention Surveys

SECTION 1: SOCIO-DEMOGRAPHIC QUESTIONS

No.	Questions	Response options
101.	How old are you?	__________ Years
102.	What is your current relationship status?	Married
		Living together with a partner
		Single
		Separated/Divorced
		Widowed
		Did not answer
103.	What is your highest level of education?	No schooling
		Primary incomplete
		Primary complete
		Secondary incomplete
		Secondary complete
		More than secondary
		Did not answer
104.	What is the main language spoken at your home?	English
		Yoruba
		Ibo
		Hausa
		Others
105.	Do you have a job for which you get paid or from which you earn money?	Yes
		No
		Did not answer
106.	In what type of dwelling or housing do you live	Brick house/apartment
		Informal dwelling/shack (e.g., in an informal or squatter settlement)
		Traditional dwelling/hut/structure made of traditional materials
		Other (please specify)____________________
		Did not answer
107.	Tell me about the place you live	
	a) Do you have electricity or a generator or a solar panel?	Yes
		No
		Did not answer

	b) Do you have tap water in your house, compound, or property?	Yes
		No
		Did not answer
	c) Do you have any type of toilet in your house, compound, or property?	Yes
		No
		Did not answer
	d) Do you or does anyone living with you have a radio?	Yes
		No
		Did not answer
	e) Do you or does anyone living with you have a television?	Yes
		No
		Did not answer
	f) Do you or does anyone living with you have internet access on a computer, a laptop or a mobile phone?	Yes
		No
		Did not answer

SECTION 2: INTRODUCTION – BREAST CANCER SYMPTOM AWARENESS MEASURE

No.	Questions	Response options
201.	Have you ever heard of breast cancer?<br>IF "NO" SKIP TO SECTION 402 = KNOWLEDGE OF SYMPTOMS	Yes
		No
202.	Do you know of any family members, friends or neighbours who have/had breast cancer?	Yes
		No

SECTION 3: KNOWLEDGE OF RISK FACTORS

301. Please could you name as many things as you can think of that could increase **any** woman's chances of getting breast cancer?"

302. Could any of the following increase **any** woman's chances of getting breast cancer?"

No	Questions	Response options
a)	Having had breast cancer previously	Yes
		No
		Don't know
b)	Drinking more than 1 bottle of beer or 1 glass of other types of alcohol per day	Yes
		No
		Don't know
c)	Not breastfeeding	Yes
		No
		Don't know
d)	Using hormone replacement therapy *[Explanation]: As women get older their hormone levels become lower and their periods stop. They may experience hot flushes and other discomforts. The medication to help with these discomforts is called hormone replacement therapy*	Yes
		No
		Don't know
e)	Wearing a tight bra	Yes
		No
		Don't know
f)	Using hormonal family planning methods (e.g., the pill, injectable contraceptives and implants)	Yes
		No
		Don't know
g)	Being overweight	Yes
		No
		Don't know
h)	Wearing a bra all the time, including at night when sleeping	Yes
		No
		Don't know
i)	Having a family member with breast cancer	Yes
		No
		Don't know
j)	Putting money in one's bra	Yes
		No
		Don't know
k)	Having the first child after the age of 30 years	Yes
		No
		Don't know
l)	Bewitched/witchcraft/evil spirits	Yes
		No
		Don't know

m)	Starting your periods **early**, before the age of 11 years	Yes
		No
		Don't know
n)	Doing little physical activity or manual labour	Yes
		No
		Don't know
o)	Aging/growing old	Yes
		No
		Don't know
p)	Putting a mobile phone in one's bra	Yes
		No
		Don't know
q)	Having no children at all	Yes
		No
		Don't know
r)	Having menopause **late**, after the age of 55 years<br>*[Explanation]: This is when a woman's period stops permanently*	Yes
		No
		Don't know
s)	Being exposed to dirty air or water	Yes
		No
		Don't know

SECTION 4: KNOWLEDGE OF SYMPTOMS

401. Please would you name as many symptoms or signs of breast cancer as you can think of?"

402. Can you tell me if you think the following could be signs of something serious or that something is wrong, such as breast ?"

No.	Questions	Response options
a)	A change in the position of the nipple<br>*[Explanation]: such as pointing up or down or in a different direction to normal* **(Picture available to illustrate)**	Yes
		No
		Don't know
b)	Pulling in of the nipple<br>*[Explanation]: Where the nipple no longer points outwards but into the breast* **(Picture available to illustrate)**	Yes
		No
		Don't know
c)	A change in the size of the nipple, not when pregnant or breast feeding	Yes
		No
		Don't know

d)	A change in the shape of the nipple, not when pregnant or breast feeding	Yes<br>No<br>Don’t know
e)	Nipple rash	Yes<br>No<br>Don’t know
f)	Discharge from the nipple, not when pregnant or breast feeding	Yes<br>No<br>Don’t know
g)	Bleeding from the nipple	Yes<br>No<br>Don’t know
h)	Fever	Yes<br>No<br>Don’t know
i)	Pain in one or both breasts, not when pregnant or menstruating	Yes<br>No<br>Don’t know
j)	A lump or thickening in the breast	Yes<br>No<br>Don’t know
k)	A change in colour of the breast skin, not when pregnant or breastfeeding	Yes<br>No<br>Don’t know
l)	Puckering or dimpling of the breast skin<br>*[Explanation]: like a dent or orange-peel appearance of the skin*<br>**(Picture available to illustrate)**	Yes<br>No<br>Don’t know
m)	A change in the size of the breast, not when pregnant or breast feeding	Yes<br>No<br>Don’t know
n)	A change in the shape of the breast, not when pregnant or breastfeeding	Yes<br>No<br>Don’t know
o)	A lump or thickening under the armpit/under arm	Yes<br>No<br>Don’t know
p)	Pain in the armpit/under arm	Yes<br>No<br>Don’t know

SECTION 5: HELP-SEEKING BEHAVIOUR

No.	Questions	Response options
501.	a) If you noticed a change in your breast or breasts, would you ignore it?	Yes
		No
		Don’t know
	b) If you noticed a change in your breast or breasts, would you try self-medication, for example get some ointment to apply from the local supermarket?	Yes
		No
		Don’t know
	c) If you noticed a change in your breast or breasts, would you tell someone close to you?	Yes
		No
		Don’t know
	d) If you noticed a change in your breast or breasts, would you visit a traditional healer?	Yes
		No
		Don’t know
	e) If you noticed a change in your breast or breasts, is there anything else you would do? (Please specify):	
502.	**ONLY FOR THOSE WHO SAID ‘YES’ IN 501d** If you noticed a change in your breast or breasts, how soon would you visit a traditional healer?	< 1 week
		1 week < 1 month
		1 month < 3 months
		3 months and above
503.	**FOR ALL WOMEN**<br>If you noticed a change in your breast or breasts, how soon would you visit the pharmacy or clinic or health centre or hospital?	Never
		< 1 week
		1 week < 1 month
		1 month < 3 months
		3 months and above

SECTION 6: CONFIDENCE SKILLS AND BEHAVIOR IN RELATION TO BREAST CHANGES

No.	Questions	Response options
601.	Do you ever check your breasts by yourself?	Yes
		No
602.	Are you confident that you would notice a change in your breasts?	Yes
		No
		Don’t know

603.	Have you ever been to see a nurse or clinical officer or doctor about a change you have noticed in your breasts?	Yes
		No
		Never noticed a change in my breasts
604.	Have you ever been to see a traditional healer about a change you have noticed in your breasts?	Yes
		No
		Never noticed a change in my breasts

SECTION 7: BARRIERS TO SEEKING MEDICAL HELP [BREAST CANCER]

"Would any of the following reasons make it difficult for you to see the nurse or clinical officer or doctor if you noticed a symptom or sign which you think may be serious, for example a change in your breast that could be cancer?"

No.	Questions	Response options
a)	I would find it difficult to go for medical help because I would be worried about wasting the nurse/clinical officer/doctor's time.	Agree
		Disagree
		Don't know
b)	I would find it difficult to go for medical help because I would be worried about what the nurse/clinical officer/doctor might find wrong.	Agree
		Disagree
		Don't know
c)	I would find it difficult to go for medical help because I would be worried about what tests the nurse/clinical officer/doctor might do.	Agree
		Disagree
		Don't know
d)	I would find it difficult to go for medical help because I am too busy or have other things to worry about.	Agree
		Disagree
		Don't know
e)	I would find it difficult to go for medical help because it takes too long to be seen at the clinic/health centre.	Agree
		Disagree
		Don't know
f)	I would find it difficult to go for medical help because I have no money for transport or the clinic/health centre charges.	Agree
		Disagree
		Don't know
g)	I would find it difficult to go for medical help because I would not feel confident about talking about my symptoms.	Agree
		Disagree
		Don't know
h)	I would find it difficult to go for medical help because I have had a bad experience in the clinic/health centre in the past.	Agree
		Disagree
		Don't know
i)	I would find it difficult to go for medical help because I would feel embarrassed.	Agree
		Disagree
		Don't know

j)	I would find it difficult to go for medical help because the nurse/clinical officer/doctor would not understand my language or culture.	Agree<br>Disagree<br>Don’t know
k)	I would find it difficult to go for medical help because my husband/partner or family member would not allow me to go.	Agree<br>Disagree<br>Don’t know
l)	I would find it difficult to go for medical help because I think that ‘if I have a disease like cancer there is no use for the nurse/clinical officer/doctor and I will die anyway’.	Agree<br>Disagree<br>Don’t know

SECTION 8: ASSESSMENT OF WOMEN AWARENESS OF BREAST CANCER SCREENING MODALITIES AND PRACTICES

1. Has anybody recommended any breast cancer screening modality for you?
   a. Yes ☐
   b. No ☐

   If No, skip to Q2
   If Yes, go to Q1(i) to Q1(ii)

   i. If yes, who did?
   a. Health worker ☐
   b. Social Media ☐
   c. Friends/Family/Colleagues ☐
   d. Mass media ☐
      i. Television ☐
      ii. Radio ☐
      iii. Newspaper ☐
   e. Clergy/Religious leader ☐
   f. Community Leader ☐
   g. Others (specify) ______________________

   ii. If yes, which?
   a. Clinical breast examination ☐
   b. Breast Mammography ☐
   c. Breast Ultrasound ☐

2. How regularly do you examine your breasts?
   a. At least once a month ☐ b. At Once in 2-6 months ☐ c. Once in 7-12 months ☐ d. Rarely ☐ e. Never ☐

3. Have you ever had your breast examined by a health practitioner?
   a. Yes ☐ b. No ☐

4. When was the last time you had such examination by a health practitioner? ______(months)

5. If recommended as a means of early detection and prevention of unnecessary death from breast cancer, will you be willing to have a health practitioner examine your breast annually?
   a. Yes ☐ b. No ☐

6. Who will you prefer to examine you?
   a. Male practitioner ☐ b. Female practitioner ☐ c. Don't care about the gender ☐ d. Don't want an examination ☐

7. Have you ever heard of mammography?
   a. Yes ☐ b. No ☐

   If No, skip to Q8
   If Yes, go to 7(i) to 7(iii)

i. If yes, what is mammography?

a. It is a blood test ☐
b. It is a urine test ☐
c. It is a form of X-ray of breast ☐
d. Don't know ☐

ii. If yes, who should have mammography?

a. Teenage girls ☐
b. Women in their twenties ☐
c. Women in their thirties ☐
d. Women forty years and above ☐
e. Only elderly women ☐
f. Don't know ☐

iii. If yes, how frequently should mammography be done?

e. Once in a life time ☐
f. Every 1 to 2 years ☐
g. Once in 5 years ☐
h. Every 10 years ☐
i. Don't know ☐

8. Have you ever had mammography done?

a. Yes ☐ b. No ☐

If No, skip to Q8(ii) to 8(iv).
If Yes, go to Q8(i)

i. If yes, when last did you have mammography done?

a. <1 year ☐
b. 1-2 years ☐
c. >2 years ☐
d. Never ☐

ii. If no to Q8, do you know where mammography can be done around you?

a. Yes ☐ b. No ☐

iii. If no to Q8, what would you say is/are the reason(s) why you have not had a mammography done before?

a. Don't know about the test ☐
b. Don't know where to do the test ☐
c. Nobody has told me the importance of the test ☐
d. Difficult to get an appointment ☐
e. I don't have the money to do the test ☐
f. Too busy to do the test ☐
g. Feel embarrassed to do the test ☐
h. Worried about what the test might show ☐
i. Don't think I need the test ☐
j. Others (please specify) _________________________________

iv. If no to Q8, which of the above is the most important reason why you have not had mammography done?

a. Don't know about the test ☐
b. Don't know where to do the test ☐
c. Nobody has told me the importance of the test ☐
d. Difficult to get an appointment ☐
e. I don't have the money to do the test ☐
f. Too busy to do the test ☐
g. Feel embarrassed to do the test ☐
h. Worried about what the test might show ☐
i. Don't think I need the test ☐
j. Others (please specify) ________________________________

9. If mammography were recommended for you as a means of preventing death from breast cancer, will you be able to afford 10,000 Naira for the test?

a. Yes ☐ b. No ☐

10. If No to Q9, how much can you afford per year? ______________________________
