# Supplementary material for: Improving access to breast cancer screening and treatment in Nigeria: The triple mobile assessment and patient navigation model (NCT05321823): A study protocol
Source: PLoS One. 2023 Jun 13;18(6):e0284341. doi: 10.1371/journal.pone.0284341 (PMC10263304; doi:10.1371/journal.pone.0284341)
Supplement: S4 File — (PDF) [file pone.0284341.s004.pdf]

Improving Access to Breast Cancer Screening and Treatment in Nigeria: The Triple Mobile Assessment and Patient Navigation Model

SOCIO-DEMOGRAPHIC CHARACTERISTICS

1. Initials: ____
2. (a) Age at enrollment: ____________ (b) Date of Birth (DD/MM/YYYY):: ___________
3. Address: ____
4. Ethnicity: Yoruba [ ] Hausa [ ] Igbo [ ] Others [ ] Specify ____________________
5. Patient's Phone No: ___________________________
6. Name next of kin: ____
7. Next of kin's phone number: ________________________
8. Marital status: Married [ ] Single [ ] Living together with a partner [ ] Separated/Divorced [ ] Widowed [ ] Did not answer [ ]
9. What is/was your most recent occupation? If married, what is/was your spouse's most recent occupation?

Occupation	You	Spouse
Unemployed	☐	☐
Pensioner	☐	☐
Civil Servant (Junior)	☐	☐
Civil Servant (Senior)	☐	☐
Trader	☐	☐
Petty Farmer	☐	☐
Cash Crop Farmer	☐	☐
Driver	☐	☐
Self Employed	☐	☐
Retired	☐	☐
Student	☐	☐
Professor / Lecturer	☐	☐
Doctor of Medicine	☐	☐
Nurse	☐	☐
Other		

10. If married, what is/was your spouse's most recent occupation? Unemployed [ ] Pensioner [ ] Civil Servant (Junior) [ ] Civil Servant (Senior [ ] Trader [ ] Petty Farmer [ ] Cash Crop Farmer [ ] Driver [ ] Self Employed [ ] Retired [ ] Student [ ] Professor/Lecturer [ ] Doctor of Medicine [ ] Nurse [ ] Other [ ]Level of formal education completed: None [ ] Primary [ ] Secondary [ ]Tertiary[ ] Postgraduate [ ]
11. Level of formal education completed: None [ ] Primary [ ] Secondary [ ]Tertiary[ ] Postgraduate [ ]
12. What type of accommodation do you live in? ☐ Single room ☐ A room and a parlour
    ☐ Two bedrooms flat ☐ 3 bedroom flat ☐ Others _________
13. House ownership status of a family: Rented from private owners [ ] Personally own house [ ] Joint ownership [ ] Government property [ ] Others specify:___________________
14. Including you, what was your <u>household's</u> AVERAGE MONTHLY income (total) over the last year? ______________(Naira) ☐ Don't know ☐ Refused
15. What was your <u>personal</u> AVERAGE MONTHLY income (including all sources) over the last year? ______________ (Naira) ☐ Don't know ☐ Refused
16. Do you have health insurance?

    ☐ Yes ☐ No

17. If yes to Q15, what type of health insurance
    Public [ ] Private [ ] Social / Community based [ ]

18. Please answer the following questions about the house you live in:

i.	Types of wall (OBSERVE)<br>1= Concrete 2= Cloth/Tarpaulin 3=Wood 4= Dirt 5= Metal<br>6= others (Please Specify____________________)
ii.	Type of floor (OBSERVE)<br>1= Concrete 2= Cloth/Tarpaulin 3=Wood 4= Dirt 5= Metal<br>6= others (Please Specify____________________)
iii.	Type of roof (OBSERVE)<br>1= Concrete 2= Cloth/Tarpaulin 3=Wood 4= Dirt/grass<br>5= Metal 6= others (Please Specify____________________)
iv.	How many of the following household Items does the respondent's household owns *(Multiple response)*<br>Bed_____, Bicycle_______, Motorcycle______, Car _____ Radio______ Television______, Mobile Phone_______ Refrigerator______
v.	How many of the following domestic animals do you own?<br>Chicken ____, Goat____ sheep____ cattle______ Other (specify______________)<br>______
vi.	Number of rooms for sleeping: ____ Average number of people per room ____________
vii.	Means of sewage (excreta) disposal<br>1= Water closet 2= pit latrine 3= VIP latrine, 4= public toilet 5= open defecation<br>6= others (specify__________________)
viii.	Means of refuse (solid waste) disposal<br>1= open dumping, 2= burning 3= Burying 4= Refuse collector<br>5= others (specify________________________________)
ix.	Sources of water for household use (multiple response)<br>1= Piped connection to yard or in household<br>2= Public standpipe 3= Borehole 4= Protected dug well 5= Protected spring 6=Rainwater 7= Unprotected dug well 8= Unprotected spring 9= Vendor water 10= Bottled water<br>11= Tanker 12= surface water sources 13. Others (specify)

19. Date of first presentation for breast cancer screening: ________________________

20. Mode of presentation:

Screening [ ] Symptomatic [ ]

If symptomatic, then tick appropriate symptom(s):

=>Pain [ ] Lump [ ] Nipple discharge [ ] Other (specify) _________

=>When did you first notice the symptom? _________________________

DETAILS OF PREVIOUS BREAST EVALUATION

21. Ever had CBE: Yes [ ] No [ ]
22. If Q17 is "yes", Report / Result of last CBE: ___________________________
    a. Normal
    b. Abnormal (Specify)______________
    c. Don't know
23. Ever had Breast Ultrasound: Yes [ ] No [ ]
24. If Q19 is "yes", Report / Result of last Breast Ultrasound: ______________________
    a. Normal
    b. Abnormal (Specify)______________
    c. Don't know
25. Ever had Breast Mammo: Yes [ ] No [ ]
26. If Q 21 is "yes", Report / Result of last Breast Mammo: ________________________
    a. Normal
    b. Abnormal (Specify)______________
    c. Don't know

BREAST CANCER RISK FACTORS

27. Age at Menarche: _____________
28. (a) Parity _____________ (b) Number children alive ___________
29. Age at first confinement ___________________________
30. Use of Oral Contraceptives Pills (OCPs): Yes [ ] No [ ]
31. Menopausal status: Pre-menopausal [ ] Perimenopausal [ ] Menopausal [ ]
32. Family history of breast cancer? Yes [ ] No [ ]
33. If yes to Q31, who (how are they related to you):

34. At what age did the person developed breast cancer __________(year) Don’t know [ ]

35. Previous personal history of breast cancer? Yes [ ] No [ ]

Anthropometry

36. Weight ______kg

37. Height _______m

38. BMI _______k/m^2

CLINICAL BREAST EXAMINATION (CBE)

(*For all categories of patients – asymptomatic, and symptomatic patients with or without an obvious lesion*)

39. Date of CBE: _____________________

40. Name of examiner: _________________

41. **Visual Exam:**

Right Breast

Skin: Normal [ ] peau d orange [ ] Dimpling [ ] Ulceration [ ] Other [ ]

Specify______________

Nipples: Everted [ ] Inverted [ ] Retraction [ ] Ulceration [ ] Discharge [ ]

Pategoid changes of the nipple-areolar complex [ ]

Left Breast

Skin: Normal [ ] peau d orange [ ] Dimpling [ ] Ulceration [ ] Other [ ]

Specify______________

Nipples: Everted [ ] Inverted [ ] Retraction [ ] Ulceration [ ] Discharge [ ]

Pategoid changes of the nipple-areolar complex [ ]

42. **Palpation:**

Right Breast: Normal [ ] Nodularity/ Lumpiness [ ] [ ] Lump/Mass [ ]

Left Breast: Normal [ ] Nodularity/ Lumpiness [ ] [ ] Lump/Mass [ ]

Location of positive findings

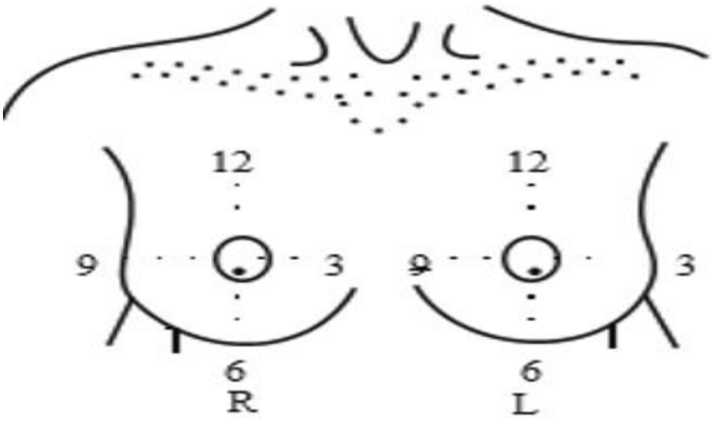

Lymph Nodes:

Left Axillary: Not palpable [ ] Palpable Mobile [ ] Palpable Fixed [ ]

Right Axillary: Not palpable [ ] Palpable Mobile [ ] Palpable Fixed [ ]

Left Supraclavicular: Not palpable [ ] Palpable Mobile [ ] Palpable Fixed [ ]

Right Supraclavicular: Not palpable [ ] Palpable Mobile [ ] Palpable Fixed [ ]

Describe all clinical findings including NORMAL and ABNORMAL (indicate size, mobility, location of palpable findings).

Findings:____

____

Plan: ____

Summary of Breast Visual inspection findings: ______________________

Summary of Breast Palpation findings: ___________________________

INNOVATIVE HANDHELD DEVICE BREAST EXAMINATION (IBE)

(*Only for asymptomatic and symptomatic patients without an obvious lesion*)

43. Date of iBE:____________________________________

44. Name of examiner: ____

45. iBE Findings:

Right breast

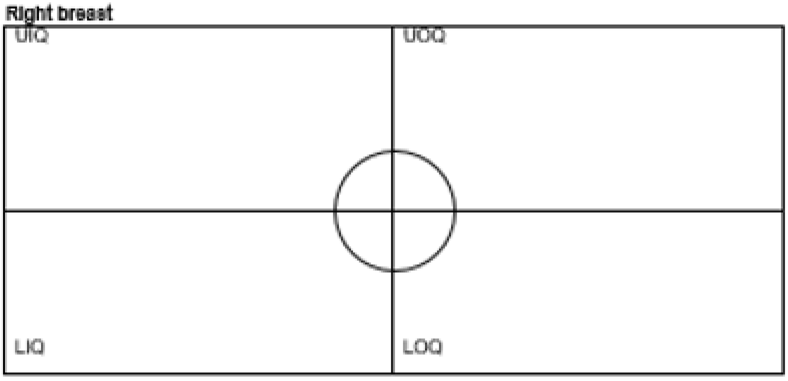

Left breast

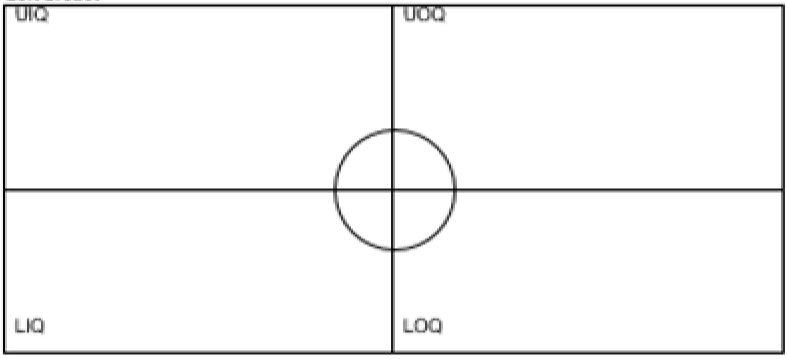

Duration of IBE examination (in minutes) __________________________

Summary of IBE findings

____

____

46. Recommendation post CBE and iBE, circle as appropriate and capture the date of further assessment:
    a. For CBE/iBE negative asymptomatic patients, repeat screening in 1 year [ ]
       Schedule date of repeat screening _____________
    b. For CBE/iBE negative symptomatic patients, repeat screening in 1 month [ ]
       Schedule date of repeat screening ______________
    c. For CBE/iBE postive (symptomatic or asymptomatic) patients, to have further imaging (USS & Mammo) [ ] Schedule date of further imaging ________________

BREAST IMAGING

Study done: Mammography ☐ Ultrasound ☐

Mammography

Study site: Study date:

Mammographic Technique:

Routine views: ☐ Special views: ☐

Breast Density:

Fatty ☐ Scattered ☐ Heterogeneously dense ☐ Extremely dense ☐

Breast findings:

Circle Positive or Negative for each quadrant, next table enables entry of details about findings

	UOQ	UIQ	LIQ	LOQ
Right Mammo	Positive or Negative	Positive or Negative	Positive or Negative	Positive or Negative
Left Mammo	Positive or Negative	Positive or Negative	Positive or Negative	Positive or Negative

Fill out one column per finding, can print additional sheets for the occasional patient with many findings.

	Finding 1	Finding 2	Finding 3	Finding 4
Laterality	Right or Left	Right or Left	Right or Left	Right or Left
Quadrant	UOQ UIQ<br>LOQ LIQ	UOQ UIQ<br>LOQ LIQ	UOQ UIQ<br>LOQ LIQ	UOQ UIQ<br>LOQ LIQ
Type of Finding	Mass<br>Calcifications<br>Distortion<br>Asymmetry	Mass<br>Calcifications<br>Distortion<br>Asymmetry	Mass<br>Calcifications<br>Distortion<br>Asymmetry	Mass<br>Calcifications<br>Distortion<br>Asymmetry
Mass Shape	Oval<br>Round<br>Irregular	Oval<br>Round<br>Irregular	Oval<br>Round<br>Irregular	Oval<br>Round<br>Irregular
Mass Margin	Circumscribed<br>Obscured<br>Micro lobulated<br>Indistinct<br>Spiculated	Circumscribed<br>Obscured<br>Micro lobulated<br>Indistinct<br>Spiculated	Circumscribed<br>Obscured<br>Micro lobulated<br>Indistinct<br>Spiculated	Circumscribed<br>Obscured<br>Micro lobulated<br>Indistinct<br>Spiculated
Mass Density	High<br>Equal<br>Low<br>Fat containing	High<br>Equal<br>Low<br>Fat containing	High<br>Equal<br>Low<br>Fat containing	High<br>Equal<br>Low<br>Fat containing
Calcification Morphology	Benign<br>Suspicious	Benign<br>Suspicious	Benign<br>Suspicious	Benign<br>Suspicious
	Micro calcification<br>Casting<br>Rod-like<br>Linear<br>Popcorn<br>Vascular	Micro calcification<br>Casting<br>Rod-like<br>Linear<br>Popcorn<br>Vascular	Micro calcification<br>Casting<br>Rod-like<br>Linear<br>Popcorn<br>Vascular	Micro calcification<br>Casting<br>Rod-like<br>Linear<br>Popcorn<br>Vascular
Calcification: Distribution	Diffuse/Regional<br>Grouped/Linear<br>Segmental	Diffuse/Regional<br>Grouped/Linear<br>Segmental	Diffuse/Regional<br>Grouped/Linear<br>Segmental	Diffuse/Regional<br>Grouped/Linear<br>Segmental
Associated Features				
Final BI-RADS	1/2/3/4/5/6	1/2/3/4/5/6	1/2/3/4/5/6	1/2/3/4/5/6

Breast parenchyma architecture: Preserved ☐ Distorted ☐

If distorted, please circle the location: RUOQ, RUIQ, RLOQ, RLIQ

LUOQ, LUIQ, LLOQ, LLIQ

Enlarged Intra-mammary lymph nodes: Yes ☐ No ☐

If Yes, circle location: RUOQ RUIQ RLOQ RLIQ

LUOQ LUIQ LLOQ LLIQ

Axillary findings:

Lymph Node: Yes ☐ No ☐

Laterality: Right ☐ Left ☐

Calcification: Absent ☐ Micro calcification ☐ Macro calcification ☐

Ultrasound

Study site: Study date:

Background tissue composition:

Homogenously fatty ☐ Homogenously fibroglandular ☐ Heterogenously fibroglandular ☐

Breast findings:

Circle Positive or Negative for each quadrant, next table enables entry of details about findings

	UOQ	UIQ	LIQ	LOQ
Right Breast	Positive or Negative	Positive or Negative	Positive or Negative	Positive or Negative
Left Breast	Positive or Negative	Positive or Negative	Positive or Negative	Positive or Negative

Fill out one column per finding, can print additional sheets for the occasional patient with many findings.

	Finding 1	Finding 2	Finding 3	Finding 4
Laterality	Right or Left	Right or Left	Right or Left	Right or Left
Quadrant	UOQ UIQ<br>LOQ LIQ	UOQ UIQ<br>LOQ LIQ	UOQ UIQ<br>LOQ LIQ	UOQ UIQ<br>LOQ LIQ
Type of Finding	Mass<br>Calcifications<br>Distortion<br>Asymmetry	Mass<br>Calcifications<br>Distortion<br>Asymmetry	Mass<br>Calcifications<br>Distortion<br>Asymmetry	Mass<br>Calcifications<br>Distortion<br>Asymmetry
Mass Echotexture	Heterogenous<br>Homogenous	Heterogenous<br>Homogenous	Heterogenous<br>Homogenous	Heterogenous<br>Homogenous
Mass Echogenicity	Hyperechoic<br>Hypoechoic<br>Isoechoic<br>Anechoic<br>Complex	Hyperechoic<br>Hypoechoic<br>Isoechoic<br>Anechoic<br>Complex	Hyperechoic<br>Hypoechoic<br>Isoechoic<br>Anechoic<br>Complex	Hyperechoic<br>Hypoechoic<br>Isoechoic<br>Anechoic<br>Complex
Mass orientation	Wider than tall<br>Taller than wide	Wider than tall<br>Taller than wide	Wider than tall<br>Taller than wide	Wider than tall<br>Taller than wide
Mass Shape	Oval<br>Round<br>Irregular	Oval<br>Round<br>Irregular	Oval<br>Round<br>Irregular	Oval<br>Round<br>Irregular
Mass Margin	Circumscribed<br>Obscured<br>Micro lobulated<br>Indistinct<br>Spiculated	Circumscribed<br>Obscured<br>Micro lobulated<br>Indistinct<br>Spiculated	Circumscribed<br>Obscured<br>Micro lobulated<br>Indistinct<br>Spiculated	Circumscribed<br>Obscured<br>Micro lobulated<br>Indistinct<br>Spiculated
Calcification: Morphology	Benign<br>Suspicious	Benign<br>Suspicious	Benign<br>Suspicious	Benign<br>Suspicious
Calcification: location	In mass<br>Outside mass<br>Intra ductal	In mass<br>Outside mass<br>Intra ductal	In mass<br>Outside mass<br>Intra ductal	In mass<br>Outside mass<br>Intra ductal
Associated Features				
Final BI-RADS	1/2/3/4/5/6	1/2/3/4/5/6	1/2/3/4/5/6	1/2/3/4/5/6

Breast parenchyma architecture: Preserved ☐ Distorted ☐

Enlarged intra-mammary lymph nodes: Yes ☐ No ☐

If Yes, circle location: RUOQ, RUIQ, RLOQ, RLIQ

LUOQ, LUIQ, LLOQ, LLIQ

Axillary findings:

Lymph Node: Yes ☐ No ☐

Laterality: Right ☐ Left ☐

Shape: Round ☐ Reniform ☐ Irregular ☐

Calcification: Absent ☐ Macro-calcification ☐ Micro-calcification ☐

Cortical thickening: Yes ☐ No ☐

If Yes: (mm): Focal ☐ Diffuse ☐

Cortical thickening in mm: ______________

Hilum: Absent ☐ poorly defined ☐ Preserved ☐

Recommendation post imaging:

a. Biopsy ☐
b. Repeat imaging in 6 months ☐ or 1year ☐
   Schedule date of repeat screening _____________

Ultrasound-guided biopsy

Core biopsy of the breast : Yes ☐ No ☐

Lymph node FNAC/biopsy: Yes ☐ No ☐

PATHOLOGY INVESTIGATIONS

47. Breast Biopsy:

Date of biopsy: _________________________

Date of histopathology report: _____________________

48. Pathology findings: Benign [ ] Malignant [ ] High risk [ ]

If malignant, then tick as appropriate:

DCIS [ ] IDC [ ] Lobular Ca [ ] Other (specify) ____________________

49. Nottingham grade_____________________

50. Immunohistochemistry ER [ ] PR [ ] HER 2 [ ]

TREATMENT

51. Date of referral to OAUTHC: ___________________

52. Date of first clinic visit at OAUTHC: ________________

53. Stage at presentation:

T stage ________ (Indicate size in centimeter) __________

N stage_________

M stage __________

54. Type of treatment commenced with date:

Neo-Adjuvant Chemotherapy [ ] Date: ____________________

Surgery [ ] Date: ____________________

55. Other treatments received with dates

Hormonal therapy [ ] Date: ____________________

Targeted therapy [ ] Date: ____________________

Radiotherapy [ ] Date: ____________________

56. Date of completion of treatment: ___________________________
