# Supplementary material for: Improving access to breast cancer screening and treatment in Nigeria: The triple mobile assessment and patient navigation model (NCT05321823): A study protocol
Source: PLoS One. 2023 Jun 13;18(6):e0284341. doi: 10.1371/journal.pone.0284341 (PMC10263304; doi:10.1371/journal.pone.0284341)
Supplement: S5 File — (PDF) [file pone.0284341.s005.pdf]

PROPOSAL/ PROTOCOL (NCT05321823)

Improving access to breast cancer screening and treatment in Nigeria: The Triple Mobile Assessment and Patient Navigation Model

Clinical area

Oncology- Breast cancer

Area of interest focus

Breast Cancer related programs with clearly defined outcome measures to improve medical knowledge and patient care, for example community outreach

Abstract summary

In Nigeria, breast cancer incidence is rising, with 80% of cases presenting at an advanced stage with poor outcomes. Patient related factors such as lack of awareness and wrong perceptions in addition to health system deficiencies such as lack of a clearly defined framework for breast cancer screening and referral are some of the major drivers of this poor outlook. Guidelines for breast cancer screening in High Income Countries have limited applicability in Low-Medium Income Countries, hence the need for innovative, resource compatible strategies to combat the negative trend.

The goal of this study is to establish a novel community-based breast cancer program to address delayed presentation and lack of access to diagnostic and treatment facilities in South-West Nigeria. It is aimed at evaluating the impact of a novel breast cancer early detection program using triple mobile assessment (innovative handheld iBreast Exam [iBE] device, mobile ultrasound, and mobile mammography) and patient navigation program in a Nigerian community.

This study aims to provide screening to asymptomatic women 40-70 years and to provide diagnostic evaluation to women presenting with breast symptoms in a community in South-West Nigeria.

The project will use a cluster randomized design with 1 community serving as the intervention arm and another community serving as the control arm. Both communities will receive breast cancer awareness and education but only the intervention community will receive screening, mobile imaging and navigation. Screening with targeted clinical history, Clinical Breast Exam (CBE), and iBE will be performed by trained Community Health Nurses in the intervention community. Women with positive CBE or iBE findings will undergo breast imaging with mobile mammography and portable ultrasound, as well as biopsy when indicated by the Radiologist who visits the community once a month, and receive navigation by the nurses to the point of care. The control population will receive breast cancer awareness without an organized screening, imaging or navigation program. Women presenting to the Primary Health Care Centers in the control community will be referred to the Teaching Hospital as per current standard of care. Record of all breast cancer cases seen in the 2 communities during the study period will be obtained. The program metrics will include screening participation rate, cancer detection rate, stage at diagnosis and timeline from detection to initiation of treatment. The stage at diagnosis and timelines from detection to treatment compared between the 2 communities will be used to assess the impact of the intervention. The study is proposed for 2 years and an initial evaluation will be done at 1 year.

This study will provide vital data to inform future breast cancer screening efforts in Nigeria through a partnership with the Ministry of Health. The results could provide a breast cancer screening model for other resource-limited countries.

Goals and objectives

Goal:

The goal of this study is to establish a novel community-based breast cancer program to address delayed presentation and lack of access to diagnostic and treatment facilities in South-West Nigeria.

Objectives:

We will conduct an implementation study aimed at evaluating the impact of a novel breast cancer early detection and patient navigation program in a Nigerian community. The program incorporates education, screening, mobile diagnosis, and patient navigation. Breast cancer screening will combine targeted clinical history, Clinical Breast Exam (CBE), and an innovative handheld device iBreast Exam

(iBE). Patients with positive findings will undergo breast imaging with mobile mammography and portable ultrasound, biopsy when indicated, and receive navigation to the point of care. The project will use a cluster randomized design with one community serving as the intervention arm and another community serving as the control arm. The objectives are:

1. To develop a routine community-based breast cancer screening/evaluation program incorporating awareness creation, use of innovative, hand-held, mobile technology iBE device, CBE, and breast imaging with mobile mammography and portable ultrasound in a selected intervention community.
2. To evaluate the impact of community-based breast imaging and biopsy with mobile mammography and portable ultrasound on the timing and stage at diagnosis of breast cancer in a selected intervention community compared to a non-intervention control community.
3. To create a referral pathway from the community to the tertiary hospital for women with positive findings in the selected intervention community and evaluate the impact on treatment timelines compared to a non-intervention control community.

The long-term objective of this study is to develop a comprehensive breast health framework to mitigate health system delays that can impact breast cancer care and outcomes in Nigerian patients. The program and lessons learned from this study may be broadly adaptable to other parts of Nigeria and to resource-limited settings generally.

Hypothesis:
We hypothesize that a community-based breast cancer program that incorporates education, screening, mobile diagnosis, and patient navigation will increase breast cancer screening participation rates, facilitate increased uptake of breast evaluation in communities with poor geographical access to standard breast imaging, reduce the incidence of advanced breast cancer cases, and shorten patients' timelines from presentation to diagnosis and initiation of treatment.

Assessment of need for the project

The burden of breast cancer in Nigeria:
Breast cancer is a major public health challenge in Nigeria with an incidence of 52/100,000[5], three-fold higher than the incidence four decades ago. Breast cancer incidence is projected to continue to increase in the coming decades.

More worrisome, however, is the pattern of presentation – up to 80% of breast cancer cases in Nigeria present in advanced stages[6, 7]. This contributes significantly to poor breast cancer mortality rates. This pattern of late-stage presentation has remained unchanged over the last three to four decades, and consequently, when combined with limited access to treatment overall, breast cancer survival in Nigeria is poor when compared to high income countries (HICs)[8-10]. Given the projected rise in breast cancer incidence, there may be an epidemic of advanced breast cancer cases in the coming years without effective interventions. In addition to some recognized biological factors accounting for aggressiveness, it is clear that correctible epidemiological and system-related factors play major roles in late-stage presentation[11].

Reasons for poor breast cancer outcome:
Reasons for poor outcomes observed in Nigeria and other low-middle income countries (LMICs) are multifactorial. However, delayed presentation and lack of access to diagnostic and treatment facilities are recognized as principal factors accounting for poor outcomes[12,13]. Both patient and system-related factors contribute significantly to delayed presentation[12]. Patient-related factors include lack of breast cancer knowledge, fear of a cancer diagnosis and treatment, use of traditional and alternative remedies, and financial constraints. Awareness creation, community education, and advocacy to address erroneous patient beliefs must therefore form the basis of any early detection program. In addition, a lack of accessible breast cancer screening and the absence of a clearly defined clinical pathway for evaluating women with breast masses, coupled with limited access to diagnostic and treatment facilities, are key system factors requiring intervention.

This project therefore aims to address these deficiencies through a comprehensive early detection program that provides community awareness and education, a community-based screening program, a mechanism for breast imaging and biopsy using mobile equipment, and a patient navigation pathway

to the point of care. Through this program, women will be well-informed about the disease and helped to develop good health-seeking habits by the availability of a screening program; those with positive findings will be promptly diagnosed and treated.

Breast cancer screening practices in Nigeria:
Nigeria, like many other LMICs, has no breast cancer screening program. While the facilities for screening (i.e., mammography units and trained personnel) are limited, studies across Nigeria auditing screening in various hospitals where mammography services are offered show very low [14, 15] uptake rates by patients. In a survey of over 2,000 women, we found very low mammography utilization rates (2.8%) and poor awareness of mammography as a screening tool[16]. While the issue of cost and access may be considered as potential limiting factors, the use of other screening modalities, such as CBE which is readily available, is also infrequent. In our published study evaluating breast cancer screening practices in a South-Western Nigerian community, the majority of women had never been screened for breast cancer, but they were willing if they were provided access to a screening program[1].

Appropriate breast cancer screening modality in Nigeria:
Currently, there are no local data to support a specific breast cancer screening model in Nigeria. While the need to develop a breast cancer screening program is obvious, developing a successful program in a resource-limited setting requires substantial logistical and cultural considerations. Although mammography is recommended as the gold standard based on data from HICs, there are personnel, financial, and infrastructural challenges that limit its applicability for routine use in Nigeria.

The number of mammography units and trained personnel available in the country are too few to feasibly provide mammography screening to all eligible Nigerian women. Primary health care centers and district hospitals which cater to the health needs of the majority of patients in the community have limited radiological services (mainly x-ray imaging). Even when accessible, the cost of mammography poses a challenge, as more than 80% of women in the community may be unable to afford annual mammography screening. This underscores the need for a cost-effective screening model tailored to the available human and infrastructural capabilities as well as patient peculiarities.

Various guidelines, such as the Breast Health Initiative Resource Stratified guidelines, have been proposed for breast cancer screening in resource-limited settings[17]. This includes the role of primary health care workers in performing CBE, creating awareness, and patient navigation for prompt treatment. Drawing from experiences of countries that have successfully demonstrated the feasibility of breast cancer screening performed by community workers using less expensive modalities, such as CBE, it is logical to build on such models[3,18].

To reduce the subjectivity of CBE, which depends on the skills of the examiner, the i-Breast Examination (iBE) device (figure 1), a hand-held device that can be used by community health personnel, has been proposed as a quantitative adjunct to CBE. The aim is to improve the accuracy of CBE while maintaining the ease and accessibility of breast cancer screening. The iBE device is an automated, battery powered, portable device designed to be used by community health nurses as a screening and triage tool. The iBE is designed to be used by a community health worker or lay person after appropriate training. In resource-limited settings, it potentially provides an easily accessible, low-cost method to assess the breast for findings that warrant further evaluation. This may help identify women who should undergo mammography and breast ultrasound in a country where these resources are limited. Initial reports on the use of the device showed encouraging results in terms of accuracy and cost effectiveness[19]. In a recently concluded study, we evaluated the usefulness of the iBE device in Nigeria; trained nurses performed breast examination on over 400 high-risk and symptomatic women using the iBE device. The device was found to be easy for operators to learn and use, well-tolerated by patients, and have high sensitivity and positive predictive value in detecting breast lesions. Preliminary results of our study showed a sensitivity of about 77% for detecting clinically important lesions when iBE is combined with CBE compared to 66% for CBE alone.

Building on our earlier results, this study proposes an early detection model that utilizes iBE and CBE as the basic breast cancer screening tools for eligible women in the community and breast imaging (mobile mammography and ultrasound) for selected women based on clinical history, CBE, and iBE findings. This pilot study could provide vital data to support wider breast cancer screening within Nigeria and other LMICs with similar population dynamics.
Triple mobile assessment:

The components of the triple mobile assessment in this proposed project are iBE, mobile portable ultrasound, and mobile mammography. We have been able to secure a mobile digital mammography machine with a non-Governmental Organization in Ile Ife, Nigeria, which will be loaned to us for the proposed two-year study period. There are two mobile ultrasounds available in the Department of Radiology of the tertiary hospital selected for this study (Obafemi Awolowo University Teaching Hospitals Complex [OAUTHC]).

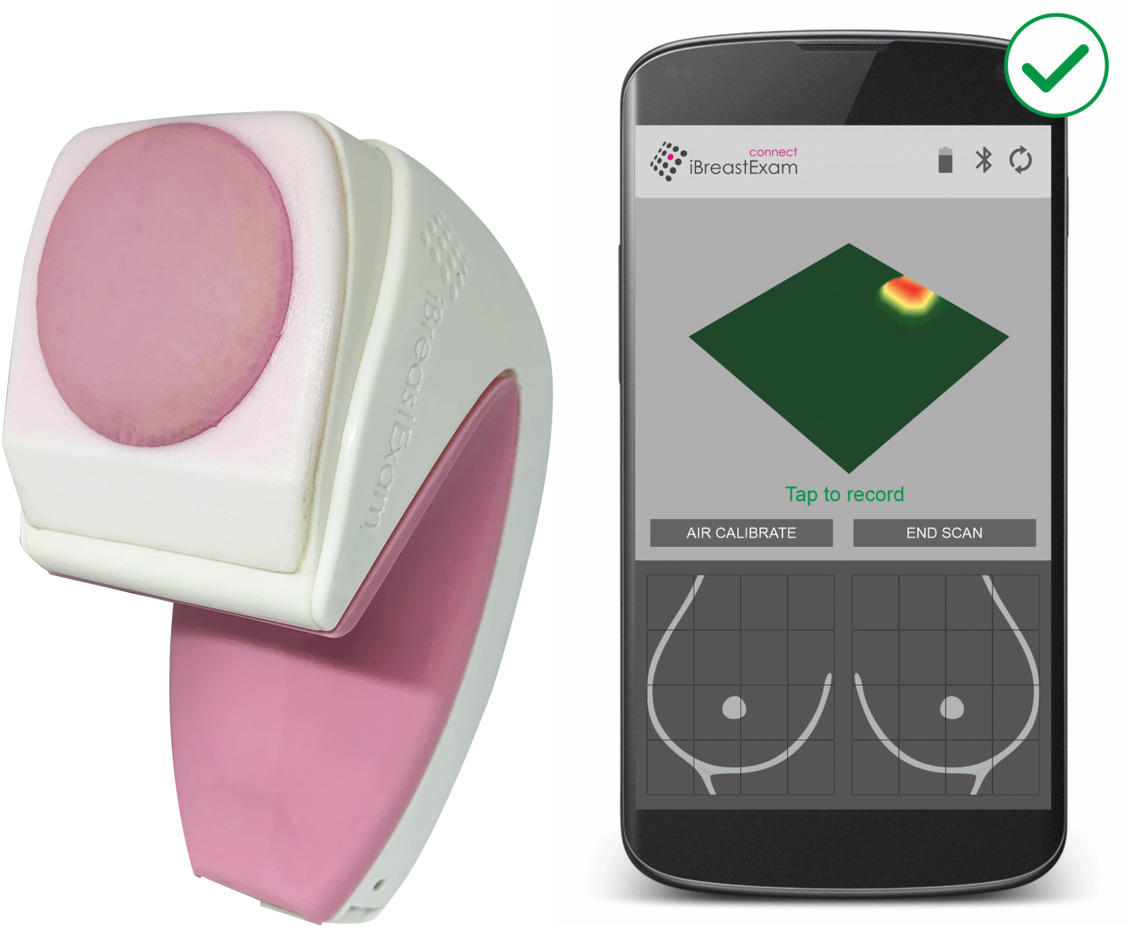

Figure 1: iBreast device (left), and sample iBE report depicting an abnormality in the breast (right).

Target population

This project aims to screen women between 40 and 70 years and to provide diagnostic evaluation on women aged 30 years and above with breast symptoms in an intervention community, Ife North Local County in Osun State, South-West, Nigeria. There are 10 districts with a population of 19,000 women in the screening age group (40–70 years) in the selected community. Three of the 10 districts with an estimated population of 5,800 eligible women will be selected. With an estimated participation rate of 70%, we project that about 4,100 women will be screened during the study period.

To screen about 4,100 participants over a year with approximately 220 working days, we estimate that a total of 19 examinations will be performed daily across the three selected Primary Health Care Centers. Three nurses per facility (9 total) will be trained to take focused breast history, perform the examinations, triage patients for imaging, and navigate patients through the referral pathway.

The control population will comprise of 2 of the 10 districts in Ife East Local County which is also located in Osun State, Nigeria. The estimated population of eligible women in 2 selected districts is about 4,500 women.

Project design and methods

Summary of study design:
This is an implementation project aimed at evaluating the impact of a breast cancer early detection and patient navigation program in a Nigerian community. The project will use a cluster randomized design, with one community serving as the intervention arm and another community serving as the control arm.

Intervention and control communities:
The study will be carried out in 2 local counties (Local Government Areas) in Osun State, South-West, Nigeria, Ife North Local County and Ife East Local County. Ife North Local County is made up of 10 districts with an estimated population of 211,000, while Ife East is divided into 10 districts with an estimated population of 259,000 (Population projection from 2006 National census).

Three districts in Ife North Local County with an estimated screening population (women 40–70 years) of 5,800 will be randomly selected to serve as the intervention community, while 2 districts in Ife East Local County with an estimated screening population of 4,500 will be randomly selected to serve as the control community. The 2 communities are similar in that they are located in the same State with similar sociocultural characteristics. Both communities are within the catchment area of the OAUTHC, which is the referral center for the 2 Local Counties. Each district is served by a dedicated Primary Health Care Center. Both communities will receive awareness and education, but only the intervention community will receive screening and navigation.

Groundwork:
To guarantee broad acceptability and aid implementation, the first phase of the project will entail interactions with the local district health authorities, traditional chiefs, women leaders, local health authorities and other opinion leaders in the community (figure 2). This project already has the support of the State Health Authorities who provided a letter of support for this project. Further consultation with the health authorities is aimed at designing an operational frame work for the utilization of the district health centers in order to ensure successful integration of the screening program into the existing health system structure of the community. Meeting with the community leaders and stakeholders is aimed at having a good understanding of the social factors that may impact on the screening program, understanding the expectations of the people and ensuring adequate community participation.

Baseline survey:
A survey of women in the 2 communities (n =163, each) will be conducted to assess baseline knowledge of breast cancer, screening practices, and perceptions about breast cancer prior to awareness creation and intervention (figure 2). Selection of women for the survey will be done using multistage sampling from the district to the street. Within each street, household sampling will be by convenience sampling of any available woman 40 years and older. The number of women sampled from each district will be proportional to the fraction of the population the district represents. Each community will be re-assessed with the same survey at the end of the first year.

Community Health Nurses will also be evaluated to assess their level of knowledge about breast cancer prior to intervention (figure 2). This will be done both quantitatively and through qualitative interviews to understand potential cultural nuances and perceptions about breast cancer that need to be factored into the execution of the project.

Training staff in the intervention community:
Community Health Nurses in the Primary Health Care Centers in 3 selected districts (n=9) will undergo a two-week training on obtaining a focused breast history, performing a CBE, use of the iBE device, and counselling (figure 2).

Training will be in the form of didactic lectures, with the use of a training manual specifically designed for this study with information derived from various sources, such as the World Health Organization

training manual and materials from the American Cancer Society. Other modes of training will include role playing, videos, and demonstrations.

Training will involve education on the basics of breast cancer, such as breast cancer epidemiology, anatomy and physiology, common benign breast conditions, breast cancer risk factors, symptoms and signs, screening, diagnosis, and treatment. The health workers will be trained on how to obtain focused breast history from women presenting for screening. The focused history includes 7 questions to assess recent breast asymmetry, breast lump, bloody nipple discharge, nipple deformity, breast skin changes, axillary swelling, and a family history of breast cancer.

Training on the performance of CBE starts with visual inspection for breast asymmetry, visible lumps, skin changes, edema, nipple retraction, discharge, or axillary swelling while the woman is in an upright position with hands on her hips and in a supine position. Health workers will subsequently be taught how to palpate the breast using the pads of the fingers with overlapping circular movements while the woman is in a supine position with the ipsilateral arm overhead. Axillary examination will also be taught. CBE lectures will be followed by video sessions, after which the technique will be demonstrated on breast models.

Training on the use of the iBE device will be conducted following the manufacturer's manual (UE Life Sciences Inc.) and led by one of the PIs of the study who has extensive experience with the device. Each health worker will be required to perform approximately 20 examinations with the iBE to achieve proficiency as noted in our pilot study.

CBE and iBE will first be performed on models, then on volunteers. After each examination, there will be a debriefing, beginning with self-evaluation, then feedback from other trainees, and then feedback from the trainers. Training is expected to last for 2 weeks based on our previous studies which demonstrated 1–2 weeks is needed to train community breast health volunteers in these exams.

Training will also include other important components such as patient counselling, effective communication, and professional ethics, such as confidentiality and data management.

After the training session, each trained health worker will give a health talk which will be evaluated by the trainers to demonstrate their ability to counsel and effectively communicate with patients.

Awareness creation and community mobilization in the intervention community
Recruitment will be done via media advertisements, posters, billboards, and community awareness programs targeting all eligible women (women between 40–70 years) to participate in screening at designated health centers and hospitals. In our previous studies, we have found these methods to be successful in meeting our recruitment goals.

a. Media advertisements
   Radio jingles in English and Yoruba, the local language in the community, will be aired on the three major radio stations that cover the local government. This will be done twice daily for a period of two weeks prior to the launch of the program and weekly subsequently, spanning the entire period of the study.
   A fifteen-minute twice weekly live local television program will also be broadcast for two weeks prior to the commencement of the program. The aim of this is to sensitize the community to the nature of the disease, remedy inaccurate notions about the disease, and highlight the benefits of screening. This will be followed by short weekly television advertorials updating the communities about the program and inviting women who are yet to be screened.

b. Community awareness and orientation programs
   Awareness programs in the form of town hall meetings will be held quarterly in various districts across the local government. This will feature talks by breast cancer survivors, key opinion leaders in the community, and medical personnel. The awareness programs will utilize the constructs of the health belief model, which addresses the various issues associated with behavior change.

c. Printed materials

Handbills and educational materials detailing the nature of the program, as well as educational materials about breast cancer and instructional details on breast examination will be distributed to women at strategic locations, such as markets and places of worship.

Screening schedule:
In the intervention community, consecutive consenting asymptomatic women (age 40–70 years) presenting for screening at designated primary health care centers will be evaluated by the trained community health nurses (n =4100). Women who are too sick to present to the Primary Health Care Centers on their own without support will be excluded from the study. Evaluation will entail a focused breast history, CBE, and iBE. Women with positive findings on CBE or iBE will be scheduled for another visit to have breast imaging and if indicated a breast biopsy. Women with negative findings on CBE and iBE will be scheduled for a similar repeat annual evaluation. Women will, however, be told to return at any time if any abnormality is noticed before their scheduled visit.

Every woman who is screened will be given a breast health card (pink card) containing the name, contact information, a summary of findings on evaluation, and date of next examination (figure 3). A duplicate copy of the breast card will be kept at the health center for reference and follow up.

In addition to the breast health card, a detailed record of CBE (figure 4) and iBE (figure 5) examination findings for each patient will be kept by the Primary Health Care Nurse.

Symptomatic women (age 30-70 years) without an obvious lesion will be evaluated in a manner similar to the asymptomatic population, with focused breast history, CBE and iBE. Those with positive findings will progress to imaging while those negative findings; will be scheduled for repeat evaluation on a short-term basis (1 month). If symptoms persist at time of next evaluation, they will progress to imaging regardless of CBE and iBE findings. Symptomatic women (age 30years and above) with obvious breast lesion(s) will undergo focused breast history and CBE examination and will be scheduled to have imaging evaluation and breast biopsy if indicated (figure 6).

Evaluation of patients with positive findings:
Women with positive CBE or iBE findings will be scheduled for imaging, which will be conducted by the Radiologist and mammography Technologist from OAUTHC who visit the community with a portable ultrasound and mobile mammography unit once a month. Patients from all the Primary Health Care Centers will be seen at a central location in the community. Patients with suspicious findings on imaging will undergo biopsy the same day. Biopsy specimens will be fixed in formalin immediately and transported to OAUTHC the same day for processing and pathologist interpretation.

Patient navigation and follow-up:
Patients with histologically confirmed breast cancer will be scheduled to visit the next available breast clinic at OAUTHC. Navigation to the breast clinic will be facilitated by the Community Health Nurses after communication of biopsy results to the patient and Nurse by the Radiologist (Figure 6).
The Community Health Nurses will also check-in with the patients regularly to ensure they comply with their appointments and follow treatment recommendations. Each patient will have a Case Report Form which will contain the CBE, iBE, imaging findings, pathology diagnosis (histology and immunohistochemistry) and their clinical data filled for them.

Control community:
In the control community, besides creating awareness about breast cancer, women are not invited to participate in a screening program and there is no community based imaging, biopsy or navigation. Women are expected to present at random to the Primary Health Care Centers if they have breast complaints which is the routine practice. Management of patients with breast related complaints will be based on the current Standard of Care which is a review by the Community Health nurse who refers or otherwise based on her assessment. Twice a month, a research staff will visit the selected Primary Health Care Centers in the control community to obtain data on the number and records of breast related cases seen at the Primary Health Care Centers and those referred to the Teaching Hospital. Patients referred to the Teaching Hospital on a suspicion of breast cancer will be tracked to determine the timelines from presentation at the Primary Health Center to eventual treatment at the Teaching Hospital and also to determine the stage at diagnosis. In addition, patients presenting directly to the Teaching Hospital from the community based on self referral will also be identified and have their data obtained.

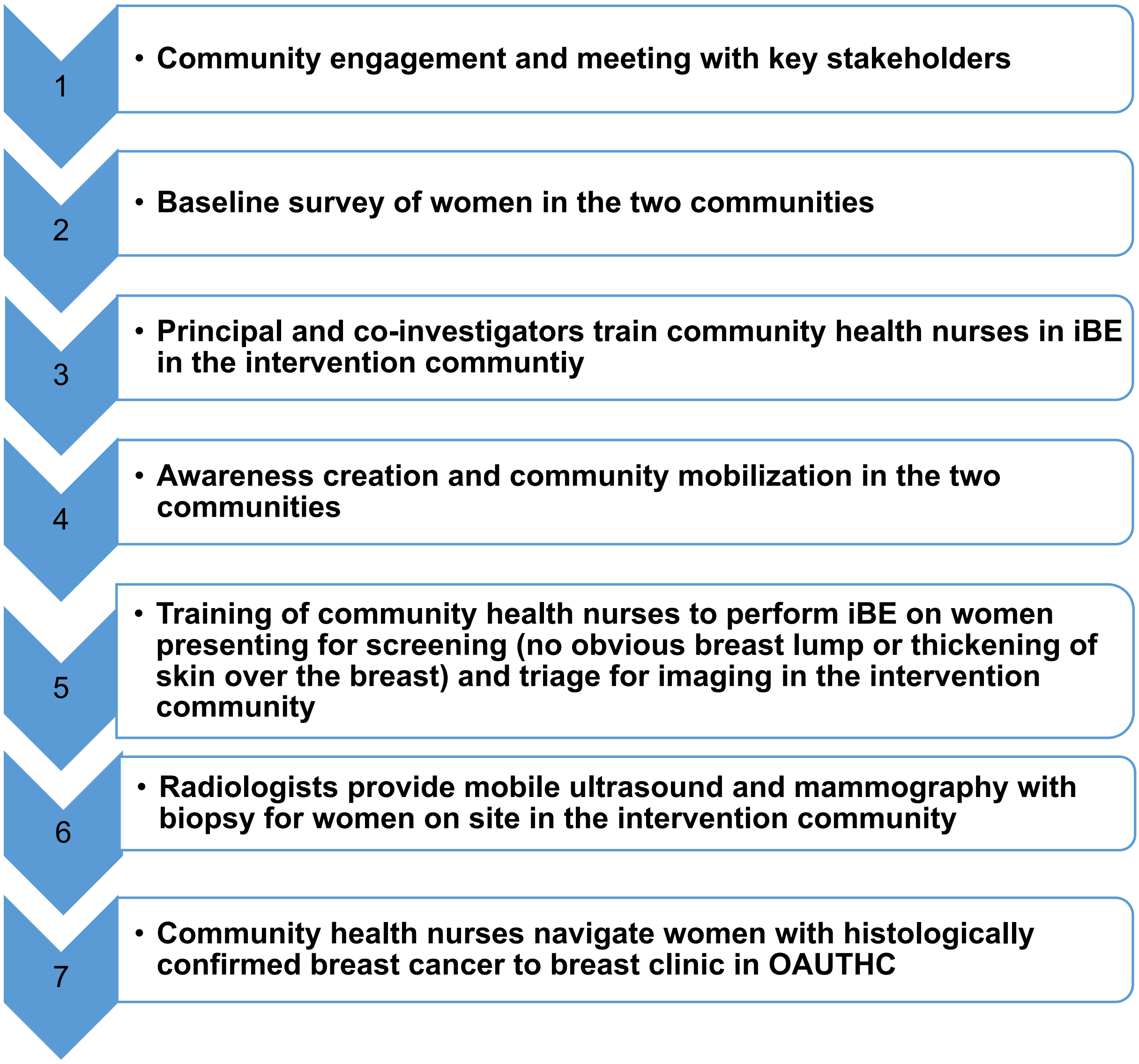

Figure 2: Summary of project design and methods

BREAST SCREENING CARD
OAUTHC

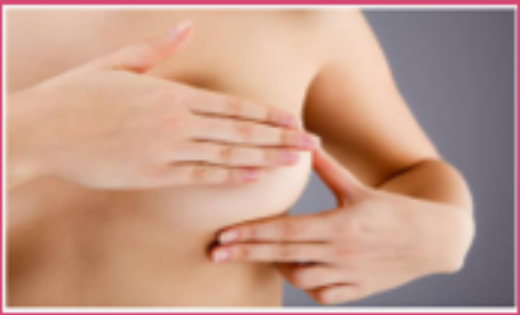

PINK CARD

Keep this card and bring it along whenever you are coming for your breast screening appointment

For Enquiries
Tel: 08021002582
08031538004
08060716670

Front page of the Breast Health card

STUDY NO: ____________

NAME: ______________________________

DATE OF BIRTH: ______________________

AGE: _______________________________

HOME ADDRESS: ______________________

____

DISTRICT: ___________________________

PHONE NO: __________________________

NEXT OF KIN'S PHONE NO: ______________

S/N	EXAM DATE	CLINIC	SUMMARY OF FINDINGS	EXAM BY	NEXT EXAM DATE

Inner page of the Breast Health Card

Figure 3: Breast Health Card

CLINICAL BREAST EXAMINATION FORM

Study no________

NAME: ______________________________ (Last / Middle / First) DOB: ________ (DD/MM/YR) DATE: ________ (DD/MM/YR)

PHONE NO: ______________________ PHONE NO. NEXT-OF-KIN: ______________________

Review of patient history

Patient noticed changes in breasts since last visit? Clinic [ ]

No Yes Describe____

Patient has a personal or family history of breast cancer?

No Yes Who ____________________ Histology diagnosis________________

Patient noted spontaneous nipple discharge? No Yes Describe ________________________

Visual Exam:

Skin: Normal Scar Dimpling Other: ________________________

Nipples: Everted Inverted Retraction

Physical Exam:

Lymph Nodes: Axillary/Clavicular Right Left

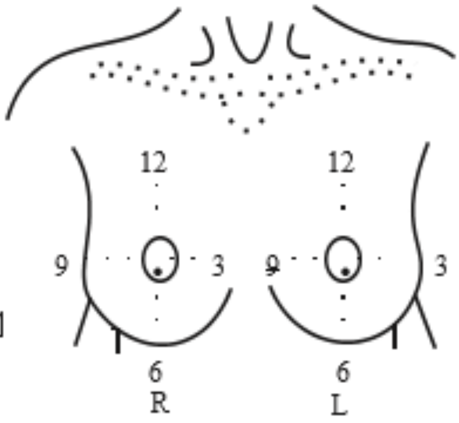

Diagram Documentation Codes:

Scar ⊤⊤⊤ Nodularity ≡ Mole *

Fibrocystic Area /// Node ○ Dimpling ⊿

Mass ●

Describe all clinical exam findings, including NORMAL and ABNORMAL (indicate size, shape, mobility, location of palpable findings).

Findings: ____

____

____

____

____

Plan: ____

Summary of Breast findings: Check one box only

☐ 1. Normal – **Re-evaluate in 1 month OR Repeat screening in 1 year**

☐ 2. Positive CBE (Mass or other findings)- **Immediate imaging**

____

Name (please print in capitals) & signature of examiner **Date**

CONTACT NUMBERS: 08021002582, 08031538004, 08060716670

Figure 4: Clinical Breast Examination form

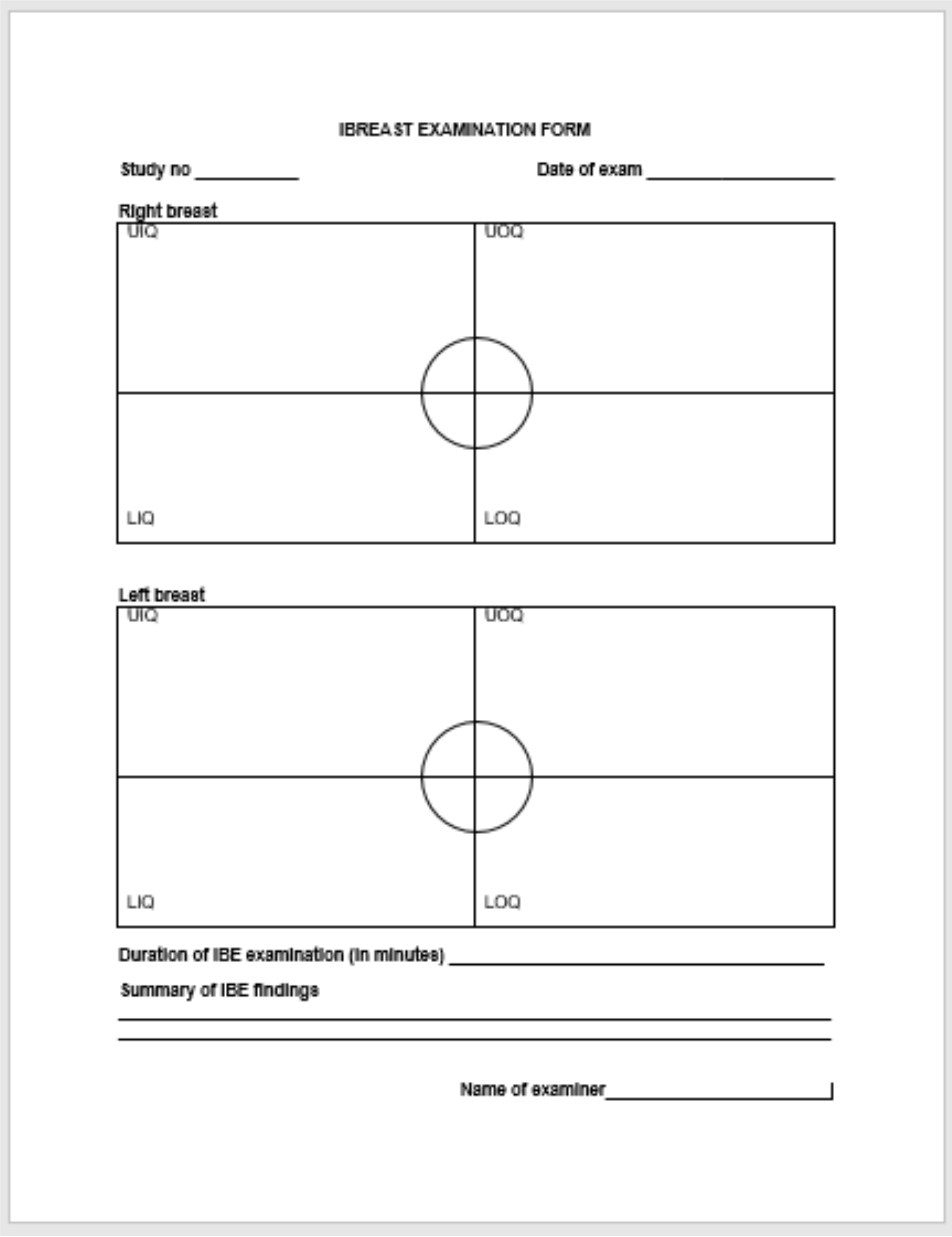
IBREAST EXAMINATION FORM

Study no ___________ Date of exam ____________________

Right breast

UIQ UOQ

LIQ LOQ

Left breast

UIQ UOQ

LIQ LOQ

Duration of IBE examination (in minutes) ________________________________

Summary of IBE findings

____

____

Name of examiner______________________

Figure 5: iBreast Examination form

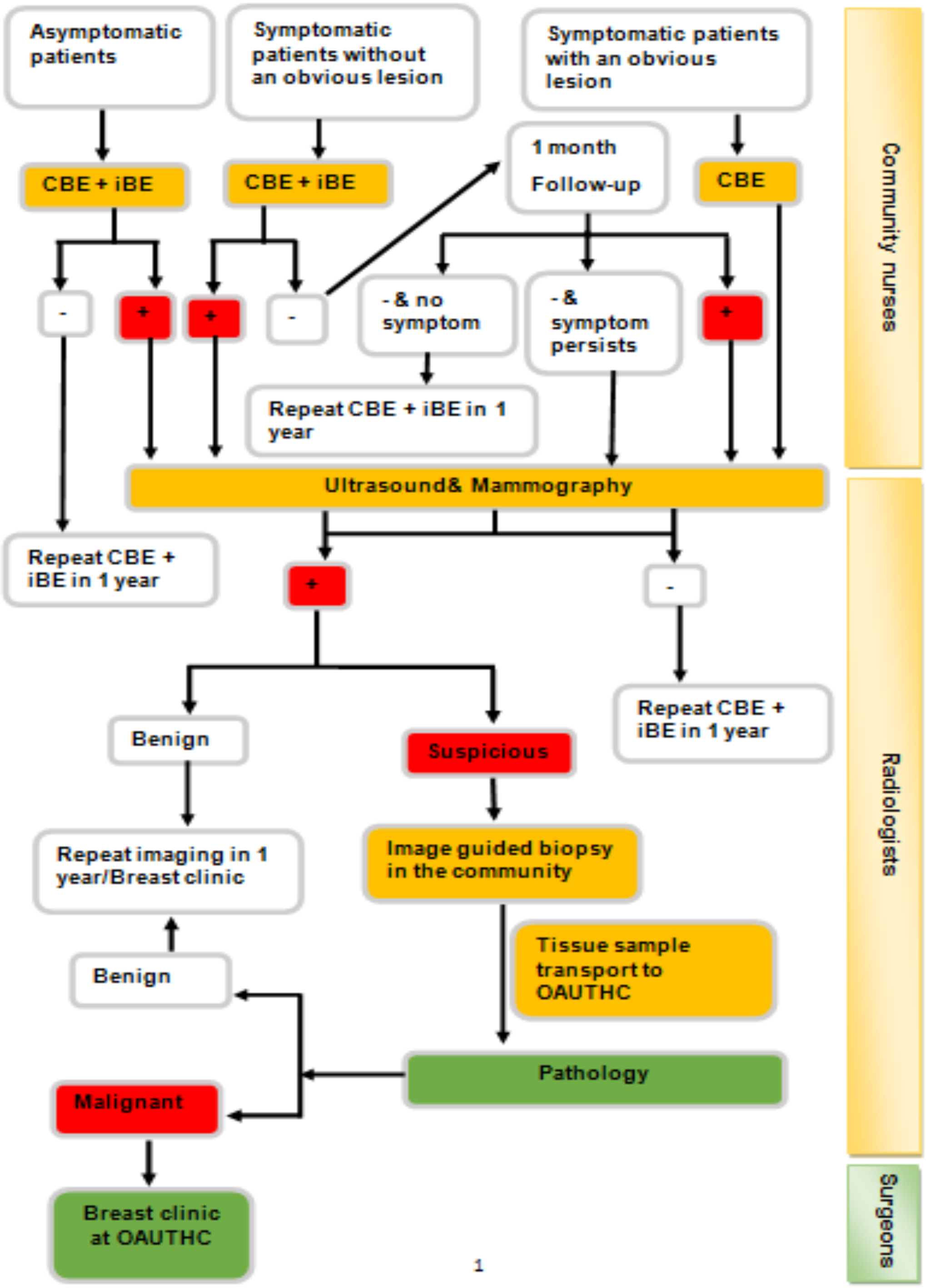

Figure 6: Navigation pathway from screening to treatment in the intervention community

Data management:
In accordance with local research regulations, data obtained by the community health nurses will be initially recorded on paper forms and stored in a secure, locked area to ensure data security and patient privacy. These data will be transferred to a REDCap database by a trained research assistant who will collect the paper data record from the various centers. All data will be collated from the centers into a central pool**.** At each center, data on the total number screened, number of those with positive findings on CBE/IBE, imaging and biopsy will be collated every two weeks. Those referred to OAUTHC will be tracked to the final point of care to determine the stage at diagnosis and to obtain information regarding treatment outcomes.

Program evaluation

The study time frame is 2 years. A review will be done at the end of year one to evaluate the progress of the research. Breast screening practices of the entire community will be re-assessed at the end of the first year. This will be compared with the baseline data already obtained prior to the commencement of the study to determine the impact of the intervention on breast cancer awareness and screening practices.

A full evaluation will be done at the end of two years. The following outcomes have been defined a priori and will be used as metrics to assess the impact of the program (figure 6).

1. Participation rate: This will be determined by calculating the percentage of women screened of the total number of eligible women based on census data. A participation rate of 70% will be considered satisfactory. The number of screened women in the intervention and control communities will also be compared.
2. Retention rate: The retention rate refers to the number of women who return for repeat annual screening of the total initial number of women screened.
3. Abnormal call rate: This is defined as the number of women with abnormalities detected on iBE and/or CBE requiring further evaluation either by imaging or biopsy out of the total number of women screened.
4. Breast cancer detection rate: This refers to the number of histologically diagnosed cases of breast cancer per 1,000 screened populations. This will be compared between the 2 communities.
5. Stage at presentation: This will be determined using both clinical and radiological assessment to determine the TNM stage among those with histologically confirmed breast cancer. It is expected that the number of late-stage diseases may be high at the outset, but this is expected to reduce as screening progresses in the intervention arm. Therefore, besides the absolute number of cases per stage, the pattern of presentation over time will also be assessed to determine if there is a decline in the number of late-stage cases. Cases of breast cancer from the intervention and control communities will be tracked both at the primary health care centers and at OAUTHC. The stage and pattern of presentation of cases from the two communities will be compared.
6. Timeline from presentation to treatment: The time interval between presentation for screening and treatment in the teaching hospital (OAUTHC) of cases from the 2 communities will be compared.

Data analysis

All data will be entered into the computer spreadsheet using Statistical Package for Scientific Solutions (SPSS) version 20 for Windows. Descriptive statistics (means, medians, variability) will be utilized to describe sociodemographic characteristics of the study participants.

Nominal categorical variables including participation rates, retention rate, abnormal call rate, and breast cancer detection rate will be compared between the control and intervention communities on contingency tables. The distributions will be compared using Chi square or Fisher's exact test (when the expected values in any of the cells of a contingency table is < 5). Ordinal variables such as stage of breast cancer at presentation will be compared between the control and intervention communities using Chi square test.

Continuous variables will be subjected to test of normality and appropriate test statistic will be used to compare the mean (for normally distributed variables) or median (for skewed distributions) between the 2 communities.

With confidence interval of 95%, p value will be set at < 0.05.

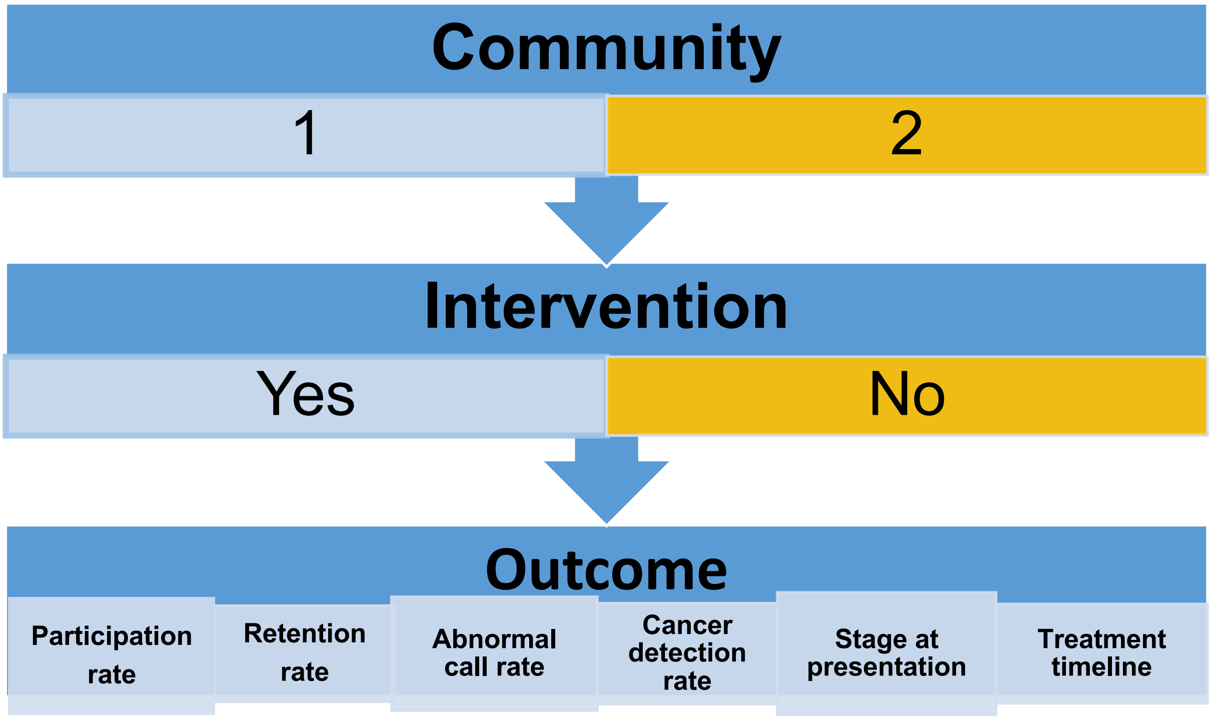

Figure 6: Outcome measures in the control and intervention communities

Expected results

We expect a participation rate of at least 70% based on figures from our baseline survey which suggests that about 70% of women were willing to participate in a screening program[1]. Going by results from breast cancer interventional programs in populations with similar patient dynamics, we project a stage shift towards early diagnosis from the current 20% to at least 50% of cases being diagnosed in early stages (stages1 & 2)[2,3]. We expect a reduction in the detection-diagnosis interval of 6.5 months to 2 months at the most[4]. This is based on a projected timeline of not more than 1 month between presentation and imaging/biopsy and not more than 1 month between biopsy and histopathological diagnosis.

Innovation

This project is the first to assess the feasibility of a broadly applicable community-based breast cancer screening program in Nigeria. The program itself is unique in that it combines the use of low-cost mobile technology and mobile imaging for routine breast screening at the community level, thereby addressing the substantial challenges of cost and geographic access to screening. Other conceptual innovations particular to this study include its aim to promote breast cancer screening as a routine practice rather than a one-time outreach activity, which can be a flaw of intervention programs. In addition, the concept of a vertical program leveraging existing health infrastructure by incorporation into an already existing health system is novel. This is a cost-effective model with applicability to many settings where funding is a challenge. Also incorporated into this study is the concept of patient navigation to the point of care for treatment and follow-up for those who require further care after screening. This study therefore provides a complete pathway – from detection to treatment – that maximizes the benefits of screening and early detection. The comprehensive design of this study, coupled with the simple but rigorous methodological approach, can generate a framework to improve breast cancer screening on a national scale.

Using a team of experts in breast cancer, community research, nursing research, radiology, and global health, we will address an unmet need for interventions to address delayed presentation and lack of access, key contributors to poor breast cancer outcomes. This project builds on our group's work in the

community to understand current screening practices, as well as barriers and perceptions regarding screening. Our findings from a community-wide survey of over 2,000 women in Osun State Nigeria demonstrate a clear need for a breast cancer screening intervention and provide the foundation for this project's design. Furthermore, we recently completed a Prevent Cancer Foundation funded project which evaluated the usefulness of the iBE device as a community screening tool. Preliminary findings show that the device is very easy to use with good sensitivity and specificity in diagnosing breast lesions, providing support for incorporation of iBE into this project. In building capacity for the performance of ultrasound-guided biopsy, which is a component of this intervention, we are currently mid-way into a National Institute of Health (NIH) funded project that is training Nigerian Radiologists in ultrasound-guided biopsies as a point-of-care procedure for the diagnosis of breast cancer. The principal investigators and co-investigators of this proposed project have an established collaborative relationship and have worked together on the aforementioned projects that are the foundation for the work proposed. (See other relevant information.)

Sustainability and scalability

We have designed this study such that it will provide information required for the next phase of the project– implementation of a routine breast cancer screening program in other parts of the country. We plan to extend the program to other institutions that are members of the African Research Group for Oncology (ARGO), an international cancer consortium involving Memorial Sloan Kettering Cancer Center (US) and several institutions in Nigeria. The simple methodology deployed in this study makes it easily reproducible in these centers, and the utilization of already existing health facilities and personnel (vertical approach) makes it easy to adopt on a large scale without significant financial and personnel requirements. The results of this study can also be utilized in the implementation of a similar screening program for other cancers of high public health interest in LMICs, such as prostate and cervical cancer.

In order to ease the implementation and integration of the results of this project into practice, relevant agencies, such as the Local Government Authority, Non-governmental Organizations are being involved in the design of the project right from inception.

Ethical approval

The study protocol is being submitted along with the ethics application form (attached) to the Ethics and Research Committee of the institution of the principal investigator (OAUTHC with international and national registration numbers IRB/IEC/0004553 and NHREC/27/02/2009a, respectively) for ethics approval which is a requirement to support our application for sponsorship from Pfizer Inc.
The protocol is also being submitted for approval from the Primary Health Care Board of the Osun State Ministry of Health.

Publication plan

The study team is committed to sharing research results as rapidly as possible among the investigators, with the cancer research community, and with relevant stakeholders. The results of our investigation will be disseminated to the scientific community via publication in international peer-reviewed journals. Our timeline and multi-pronged approach to breast cancer screening are favorable for generating multiple publications, including initial results, follow-up impact on screening, and long-term implications. Findings from this project will also be disseminated to all stakeholders in Osun state and in Nigeria as a whole. Our results will be shared with the State Commissioner of Health with the intent of disseminating it for wider implementation across the State. Our findings will also be submitted to the Nigerian National Cancer Control Committee for consideration in the National Cancer Control Plan. The African Organization for Research and Training in Cancer (AORTIC) is a veritable platform for disseminating results of research projects relevant to LMICs, it is our goal to present our findings at the biennial AORTIC conference. Our findings will also be presented at other local and international conferences, such as the West African College of Surgeons Conference, Conference of the Breast Imaging Society of Nigeria and the American Society of Clinical Oncology. This project will utilize the existing ARGO platform ARGO for dissemination and implementation of the results of this project.

<u>Anticipated project timeline</u>

Phase I: Preliminaries
Consultations with Government Health authorities
Consultations with traditional rulers, chiefs, market women, and key opinion leaders
Selection of screening centers

Phase II: Mobilization of resources, personnel, and participants
Procurement of materials
Training of personnel
Awareness creation
Community mobilization

Phase III: Implementation
Baseline assessment
Training of community health nurses
Recruitment
Data collection
Follow-up
Presentation of preliminary report at conferences

Phase IV: Completion phase
Complete follow-up
Complete data analysis
Manuscript writing
Dissemination

Milestone/Activity	Status	Expected time to completion	Is a deliverable?
• Consultations with Government Health authorities<br>• Consultations with the traditional rulers, chiefs, market women, and key opinion leaders<br>• Selection of screening centers	Started	2 Months	Yes
• Awareness creation, Mobilization of resources, personnel and participants	Not started	3 Months	Yes
• Training of personnel	Not Started	2 Weeks	Yes
• Recruitment<br>• Data collection<br>• Follow-up	Not Started	18 Months	Yes
• Manuscript writing<br>• Dissemination	Not Started	2 Months	Yes

Budget

Budget period: 11/01/2020 – 31/10/2022 (2 years)

Personnel	Appoint ment Type	% Effort	Person Months	Annual Salary ($)	Proposed Salary ($)	Fringe benefits ($)	Total amount ($)
**Key personnel**							
PI	12 months	20%	2.4	20,000	4,000	720	4,720
PI 2	12 months	20%	2.4	20,000	4,000	720	4,720
**Other Personnel**							
Project coordinator		50%	12	5,000	5,000	1800	6,800
Research assistant		100%	12	4,000	4,000	560	4,560
Research assistant 2		100%	12	4,000	4,000	560	4,560
**SUBTOTAL/YEAR**							**25,360**
**SUBTOTAL (2 YEARS)**							**50,720**

**ITEM (X 2 YEARS)**	**Amount**
**Start-up costs for town hall meetings and awareness programs**<br>Program advertisement on radio and Television<br>Facility/equipment rentals<br>Educational/instructional materials<br>Participant incentives	**2,500**
**Start-up costs - Materials and supplies**<br>4 desk top computers, 2 lap tops, Printers, UPS, Scanners, Thumb drives<br>Projectors and projector screens<br>Telephones/Air time recharge	**5,000**
**Travel**<br>**Local**<br>PI travel for consultations with State and health authorities<br>PI travel for presentation of results at local conferences<br>**International**<br>PI travel for presentation of results in 2 conferences	**2,000**<br><br>**5,000**
**Training and reimbursement of personnel**	**5,000**
**Renting of 6 i-Breast devices**	**12,000**
**Maintenance of the mobile ultrasound unit**	**2,000**
**Renting of Mobile Mammography Unit, Transportation to and from the community (Once monthly), consumables**	**30,000**
**Supplies and consumables**<br>Procurement of biopsy materials	**3,500**
**Histology and Immunohistochemistry**	**1,500**

**Treatment support for confirmed cancer cases (Surgery, chemotherapy)**	**7,500**
**Bio-statistics**	**1,000**
**Publication/ Dissemination of information**	**2,000**
**TOTAL**	**129,720**
**10% Institutional overhead**	**12,972**
**GRAND TOTAL**	**142,692**

<u>Budget justification</u>

Key Personnel:

Adeleye Omisore, MD (Principal Investigator - 20% effort).
Dr. Omisore is a radiologist at OAUTHC. Her career is focused on breast and GI radiology. She is a MPI on a protocol to train Nigerian radiologists to perform ultrasound-guided breast biopsies, a novel training program in Nigeria. She was co-investigator on a protocol evaluating the usefulness of the iBE device in Nigeria. For this current project, Dr. Omisore will coordinate the breast imaging and biopsy components of the protocol, coordinate the activities of the community health nurses and participate in their training. Dr. Omisore will provide 4.8 person months and requests salary support at 20% for the study period.

Olalekan Olasehinde, MD (Co-Principal Investigator - 20% effort)
Dr. Olasehinde is a general surgeon with a particular clinical and research interest in early diagnosis of breast cancer in Nigeria. He has expertise in health services research, and has led studies examining barriers to breast cancer screening in Nigeria – work that forms the basis and has critically informed this application. Dr. Olasehinde will coordinate various aspects of the project. He will undertake all the necessary groundwork and consultations prior to formal commencement of the screening program. He will also oversee staff selection and training and coordinate care of patients referred to OAUTHC. Dr. Olasehinde will provide 4.8 person months and requests salary support at 20% for the study period.

Olusegun Isaac Alatise, MD (Co-Investigator/Mentor - 10% effort).
Dr. Alatise is a surgeon at OAUTHC who specializes in oncology in Nigeria and West Africa and is co-founder of the ARGO consortium. He has successfully led several studies exploring disparities and community-based screening projects for cancer in Nigeria. He will provide this expertise to guide the design and conduct of this project in Nigeria. He will work closely with Dr. Olasehinde and Dr. Omisore to advise on recruitment, ethical conduct, data analysis, and dissemination of results. He will assist with all troubleshooting to ensure timely completion of the objectives. No salary support is requested.

Dr Olusola Famurewa, MD (Co-investigator-10% effort)
Dr Famurewa is a consultant Radiologist at OAUTHC whose primary area of research is in breast imaging. She will participate in the interpretation of mammograms acquired during this project. No salary support required.

Funmilola Wuraola, MD (Co-Investigator - 5% effort)
Dr. Wuraola is a surgeon at OAUTHC who is currently researching modalities for improving financial access to cancer care. She will coordinate the patient navigation program, as well as oversee the awareness and educational programs. No salary support is requested.

T. Peter Kingham, MD (Co-Investigator/Mentor)
Dr. Kingham is the Director of Global Cancer Disparity Initiatives at Memorial Sloan Kettering Cancer Center (MSK). His research focuses on defining disparities in cancer care, cancer outcomes, surgical oncology training, and introducing new diagnostic tests in low- and middle-income countries. He has successfully led several prospective studies exploring disparities and community-based screening projects for colorectal cancer in Nigeria. He will provide his expertise to guide the design and conduct of this project; he will work closely with Dr. Olasehinde and Dr. Omisore to advice on study design, recruitment, ethical conduct, data analysis, and dissemination of results. Dr. Kingham will travel to Nigeria three times during the study period for in-person meetings, and will participate in conference calls as needed. He will assist with all troubleshooting to ensure timely completion of the objectives. He will contribute to dissemination of results and guide future implementation of findings in this region. No salary support is requested.

Victoria Mango, MD (Investigator - 5% effort).
Dr. Mango is an Assistant Attending Radiologist at MSK. Her research focuses on developing and evaluating novel approaches to breast cancer imaging, with an emphasis on global clinical impact. She has expertise in global breast imaging research and education and works closely with the MSK Global Cancer Disparities Initiatives Program, the Society of Breast Imaging (SBI) International Education Outreach Committee, and RAD-AID international. Dr Mango worked as PI on the protocol for the evaluation of the iBE device in Nigeria. Dr. Mango will provide her expertise in the design and execution of the project, particularly in the training of community health nurses on the use of the iBE device; she will also coordinate with Dr. Omisore on the design of the breast imaging protocol of the project. No salary support is requested.

Anya Romannoff, MD (Investigator - 5% effort).
Dr. Romanoff is an Assistant Professor in Breast Surgical Oncology as well as the Department of Health System Design and Global Health at the Icahn School of Medicine at the Mount Sinai Hospital in New York, NY. She has pursued global health research opportunities in an effort to improve access to high-quality breast cancer care worldwide. She was a recipient of a Fulbright-Fogarty Postdoctoral Research Award in Public Health which she used to design and execute a research project to investigate access to breast cancer care in Peru. She also collaborated on an invited publication in The Lancet Oncology on resource-stratified breast cancer care. She will provide her expertise to advise on study design, study conduct, and future studies. No salary support is requested.

Margaret Barton-Burke, PhD (Investigator - 5% effort)
Dr. Barton-Burke is an Oncology Nurse with several years of experience in Nursing Oncology research, and is the Director of Nursing Research at MSK. Her current research focus is on culturally-appropriate interventions, outreach, and programs for the community of color. She currently has a breast cancer grant from Pfizer global. She will bring in her expertise in the training of the community Nurses and in the implementation of the project in general. No salary support is requested.

Elizabeth Sutton, MD (Investigator - 5% effort)
Dr. Sutton is an Assistant Attending Radiologist and Director of Breast MRI at MSK. Since 2017, she has been a member of the Global Cancer Disparities Initiatives at MSK. She led the conceptualization and design of the National Institute of Health funded capacity building project to train Nigerian radiologists to perform ultrasound-guided biopsy and is co-PI on that project with Dr. Omisore. Dr. Sutton will also be coordinating with Drs. Omisore and Mango on the design of the breast imaging and ultrasound-guided breast biopsy protocol of this project. No salary support is requested.

Other Personnel:

Project coordinator: Dr. Israel Owoade is a Specialist Public Health Physician and will serve as the project coordinator. He possesses excellent experience in community field work and data management. He will oversee the day-to-day project activities, coordinate the research assistant, and oversee data management. He will also serve as the data manager. He requests salary to support his 50% effort.

Research assistants: Salary support is requested for 2 research assistants (one research assistant to cover a community) whose responsibility will be to carry out all secretarial work, collate and enter data from all study sites under the supervision of the project coordinator, and other responsibilities that may

arise in the course of the study within the limits of their expertise. Two college graduates who will receive salary for a 12-person month contribution for the entire period of the study will be required**.** The calculation of salary is based on the current minimum wage for college graduates in Nigeria.

Start-up costs for town hall meetings and awareness program: In order to ensure support from local and state authorities as well create breast cancer screening awareness for this study, we require funds for program advertisement on radio and television, educational materials and incentives for participants. We will require $2,500 for these.

Start-up costs for Materials and supplies and awareness program: For proper onsite collection and storage of data which is key to the success of the project, a desktop computer with other accessories will be required at all the screening sites. Being a field project in community health centers where these materials were not previously available, they will need to be provided by funds from this project. We propose a system unit for each of the three screening centers and one unit for central administration. In addition to this, a laptop will be procured for use by the project coordinator.

Telephones are required for each of the screening units to facilitate communication among the various units and the central administrative office and also for communication with participants and sending reminders for screening.

We also request costs for the purchase of two projectors and two large projector screens to be used during awareness programs and for training sessions. Procuring two sets allows us the flexibility of running two events simultaneously if required. In addition, the use of audiovisuals being an important element of this project, a second unit serves as a backup. We require $5,000 for these.

Travel: A sum of $7,000 requested for travel, including both domestic ($2,000) and international ($5,000). Domestic travel costs will cover consultations with the authorities of Local, State, and Federal Ministry of Health in order to obtain all necessary approval and for the implementation of the project. The domestic travel will also include attendance at the Nigerian Surgical Research society and Breast Imaging Society of Nigeria conferences where the results of this project will first be presented.
International travel will be for presentations at two conferences, namely the American Society of Clinical Oncology conference in the United States and the conference of the African Organization for the Research and Training in Cancer.

Training and reimbursement of personnel: This project will require two-week training for the community health nurses who will carry out the examinations. For this purpose, breast models will be procured, educational and instructional materials will be provided and the participants will be reimbursed. We expect to train an average of 3 community health nurses per center, amounting to 9 people to be catered for during the training.

Equipment: Six iBreast devices will be rented for this study from the manufacturer, Life Sciences, Inc. at a cost of $1,000 per device per year. $12,000 is requested to cover this expense for the 2-year period. A mobile Mammography unit will be rented from non-Governmental Organization here in Ile Ife at the cost of $12,500 per year.

We also require $2,500 per year for maintenance and logistics for the mammography machine. An additional cost of $1,000 per year will be required for the maintenance of the OAUTHC mobile ultrasound unit, which has been made available for this project.

Supplies and Consumables (Procurement of biopsy materials): We require $3,500 for biopsy supplies which include but are not limited to biopsy needles, scalpels, skin sterilization agent, formalin for the ultrasound guided breast biopsies when indicated.

Payment for histopathology services: We require $1,500 payment for the histology and immunohistochemistry of the core biopsy samples taken for pathology in the teaching hospital (OAUTHC).

Treatment support for confirmed cancer cases: The histologically confirmed cancer cases will require treatment in the Teaching Hospital. We request $7,500 to cover the cost of surgery and chemotherapy for the confirmed cancer cases for the entire study period.

Biostatistics: We request $1000 for consultancy for the analysis of data generated from this study.

Publication and dissemination of information: We request $2,000 to cover costs associated with publications stemming from this work.

Additional information

Letters of support from our mentors and co-investigators as well as institutional and Local Health authority supports are uploaded with this application.

Organization detail

Obafemi Awolowo University Teaching Hospitals Complex (OAUTHC) Ile-Ife, Nigeria is one of the first Teaching Hospitals established by the Federal Government of Nigeria in 1972 to provide quality healthcare to its people. In 1967, the defunct Western State Government of Nigeria resolved to build a medical school at the University of Ife (which was then five years old) to provide manpower to tackle health problems. After a period of careful planning, the Faculty of Health Sciences and medical school were created on 8th May, 1972, which is now Obafemi Awolowo University (OAU).

Ife Philosophy/Concept

The Ife University Teaching Hospitals Complex (Now OAUTHC) is unique in that it was founded on what is generally referred to as the Ife Philosophy – its guiding principle. The philosophy focuses on an integrated healthcare delivery system with emphasis on comprehensive healthcare service based on a pyramidal structure, comprising primary care at the base, and secondary and tertiary services at hospital settings. This was designed to secure improvement in the physical, mental, and socio-economic well-being of Nigerians through preventive, promotive, diagnostic, restorative, and rehabilitative services. The philosophy has led to a unique way of delivering healthcare and training health professional in Nigeria.

Primary healthcare is provided to the community in its three health centers – two urban and one rural in its catchment areas at Ife, Ilesa and Imesi–Ile respectively. Secondary and tertiary level care is provided at its three major hospital facilities, in Wesley Guild Hospital Unit Ilesa, Ife State Hospital unit Ile Ife and Dental Hospital located at Obafemi Awolowo University, Ile-Ife. OAUTHC has recognized and established primary healthcare as an integral part of the livelihood and well-being of Nigerians. OAUTHC was ahead of the WHO in its Alma Ata Declaration of 1978 that primary healthcare is the key to attaining Health for all by the year 2000 and beyond.

The Ife philosophy has, thus, resulted in OAUTHC having multiple hospital units. While the Teaching Hospitals Complex, as a dynamic institution, has undergone various changes in its government, administration, management, physical resources, and service load during its 25 years of existence, its founding philosophy, the concept of provision of comprehensive health care based on integrated, primary, secondary, and tertiary healthcare delivery, has remained constant. Currently, it provides these services through six healthcare units:

- Ife Hospital Unit, Ile-Ife
- Wesley Guild Hospital, Ilesa
- The Dental Hospital, OAU, Ile-Ife
- Urban Comprehensive Health Centre Eleyele, Ile-Ife
- Rural Comprehensive health Centre, Imesi-Ile
- Multipurpose Maternal and Child Health Centre, Ilesa

Change of corporate name

The initial corporate name of the Institution as Ife University Teaching Hospitals Complex was changed to Obafemi Awolowo University Teaching Hospitals Complex in 1987 in honor of the late distinguished; elderly statesman Chief Obafemi Awolowo, who died that year.

Objectives
The cardinal objective of OAUTHC has remained essentially unchanged:
• To provide effective quality healthcare delivery to all categories of health care consumers most efficiently.
• To train/educate different cadres of health care professionals/providers for the Nigerian Nation in particular and the world in general.
• To carry out medical/health systems research for the advancement of health system knowledge for the overall purpose of raising health status of Nigeria and world citizens.
• To participate in the community health promotion to secure improvements in physical, mental and wellbeing of the people.
• To reduce the urban-pull syndrome among health professionals/providers by training them in rural setting.

Catchment area
By virtue of its location and the scarcity of health care facilities in neighboring areas, the catchment area of the OAUTHC is extremely large, including the whole of Osun, Ekiti, and Ondo State and some parts of Oyo, Kwara, Kogi, Lagos, and Edo state. While the primary base is the Ife/Ijesa Senatorial District, the institution provides tertiary, secondary, and primary health care services to the area mentioned above, which is estimated to be for about 15 million people.

Research capacity
OAU is a leading academic institution in the country that has being involved in several international and national research grants which include the Carnegie Corporation, the Bill and Melinda Gates Foundation, and the International Foundation of Science. The institution also hosts the Central Laboratory and Center of Energy Research and Development which has a number of state-of-the-art equipment that offers services to the West African sub region. It also hosts the National Center for Technology Management.

OAUTHC Leadership
The OAUTHC is headed by the Chief Medical Director, a consultant radiologist and a Professor of Radiology.

The Chief Medical Director**:** The Chief Medical Director is the Hospital's Chief Executive Officer and, in that capacity, has responsibility for the day-to-day management of the entire hospital. He is directly responsible to the Board of Management usually instituted by the President of the Federal Republic of Nigeria. He is the Chief image maker and also the Chief Accounting Officer of the institution.

Chairman, Medical Advisory Committee/Director of Clinical Services: The Chairman, Medical Advisory Committee coordinates the activities of the hospital's training schools/programs and the clinical departments. The Directorate of Clinical Services and Training is the administrative arm headed by the Chairman, Medical Advisory Committee. The Directorate oversees all clinical services and training rendered by the hospital and also supervises all clinical departments/units.

The Director of Administration: The director of Administration is the Secretary to the Management Board of the Teaching Hospital. The Management Board is a statutory body appointed by the Federal Government for the overall supervision and policy formulation of the Teaching Hospital. The office of the Director of Administration oversees non-clinical departments/units.

Breast cancer Research grants has received in the past five (5) years in OAU/OAUTHC

1. GC238015: Prevent Cancer Foundation, USA- Feasibility of breast cancer screening in high risk Nigerian women using novel low cost device
2. NCT03879577: University of Chicago Medical Center/Breast Cancer Research Foundation seed grant, USA-Assessing REsponse to neoadjuvant Taxotere and TrAstuzumab in Nigerian women with Her-2 positive breast cancer (ARETTA)
3. 1R21CA239784-01 Fogarty International Center (FIC)/ National Institute of Health /National Cancer Institute (NCI), USA- Tablet-based mobile Health Ultrasound for point-of-care breast cancer diagnosis in Nigeria

Pertinent breast cancer research landmarks demonstrating tangible outcomes in OAU/OAUTHC

1. Developing a breast cancer database and Biobank.
2. Hosting and organizing the first National Cancer Consortium meeting in Colorectal and Breast Cancer and training of over 100 doctors in colorectal and breast oncology since 2012.

Bibliography

1. Olasehinde O, Boutin-Foster C, Alatise OI, Adisa AO, Lawal OO, Akinkuolie AA et al. Developing a Breast Cancer Screening Program in Nigeria: Evaluating Current Practices, Perceptions, and Possible Barriers. *J Glob Oncol.* 2017; 3: 490-496.

2. Matovu A, Scheel JR, Shadrack PA, Ssembat R, Njeri A, Galukan M et al. Pilot study of a resource-appropriate strategy for downstaging breast cancer in rural Uganda. *J Glob Radiol.* 2016;2. DOI: 10.7191/jgr.2016.1021.

3. Ngoma T, Mandeli J, Holland JF. Downstaging cancer in rural Africa. *Int J Cancer.* 2015;136:2875-2879.

4. Agodirin O, Olatoke S, Rahman G, Olaogun J, Kolawole O, Agboola J et al. Impact of Primary Care Delay on Progression of Breast Cancer in a Black African Population: A Multicentered Survey. *J Cancer Epidemiol.* 2019;2019. DOI: 10.1155/2019/2407138

5. Jedy-Agba E, Curado MP, Ogunbiyi O, Oga E, Fabowale T, Igbinoba F et al. Cancer incidence in Nigeria: a report from population-based cancer registries. *Cancer Epidemiol.* 2012;36:e271-278.

6. Adesunkanmi AR, Lawal OO, Adelusola KA, Durosimi MA. The severity, outcome and challenges of breast cancer in Nigeria. *Breast*. 2006;15:399-409.
7. Anyanwu S. Breast cancer in eastern Nigeria: a ten year review. *West Afr J Med.* 2000;19: 120-125.

8. Altekruse S, Kosary C, Krapcho M. SEER cancer statistics review, 1975–2007. Bethesda, MD: National Cancer Institute; 2010 (based on November 2009 data submission). seer. cancer. gov/csr/1975_2007. Accessed March.2011;4.

9. Edwards BK, Noone AM, Mariotto AB, Simard EP, Boscoe FP, Henley SJ et al. Annual Report to the Nation on the status of cancer, 1975-2010, featuring prevalence of comorbidity and impact on survival among persons with lung, colorectal, breast, or prostate cancer. *Cancer.* 2014;120:1290-1314.

10. Makanjuola SB, Popoola AO, Oludara MA. Radiation therapy: a major factor in the five-year survival analysis of women with breast cancer in Lagos, Nigeria. *Radiother Oncol.* 2014;111:321-326.

11. Dietze EC, Sistrunk C, Miranda-Carboni G, O'Regan R, Seewaldt VL. Triple-negative breast cancer in African-American women: disparities versus biology. *Nat Rev Cancer.* 2015;15:248-254.

12. Ezeome ER. Delays in presentation and treatment of breast cancer in Enugu, Nigeria. *Niger J Clin Pract.* 2010;13:311-316.

13. Ibrahim NA, Oludara MA. Socio-demographic factors and reasons associated with delay in breast cancer presentation: a study in Nigerian women. *Breast.* 2012;21:416-418.

14. Akande HJ, Olafimihan BB, Oyinloye OI. A five year audit of mammography in a tertiary hospital, North Central Nigeria. *Niger Med J*. 2015;56:213-217.

15. Obajimi MO, Adeniji-Sofoluwe AT, Oluwasola AO, Adedokun BO, Soyemi TO, Olopade F et al. Mammographic breast pattern in Nigerian women in Ibadan, Nigeria. *Breast Dis*. 2011;33:9-15.

16. Olasehinde O, Alatise OI, Arowolo OA, Mango VL, Olajide OS, Omisore AD et al. Barriers to mammography screening in Nigeria: A survey of two communities with different access to screening facilities. *Eur. J. Cancer Care.* 2019;28:e12986.

17. Anderson BO, Yip CH, Ramsey SD, Bengoa R, Braun S, Fitch M et al. Breast cancer in limited-resource countries: health care systems and public policy. *Breast J.* 2006;12:S54-69.

18. Abuidris DO, Elsheikh A, Ali M, Musa H, Elgaili E, Ahmed AO et al. Breast-cancer screening with trained volunteers in a rural area of Sudan: a pilot study. *Lancet Oncol.* 2013;14:363-370.

19. Somashekhar S, Vijay R, Ananthasivan R, Prasanna G. Noninvasive and low-cost technique for early detection of clinically relevant breast lesions using a handheld point-of-care medical device (iBreastExam): prospective three-arm triple-blinded comparative study. *Indian J Gynecol Oncol*. 2016;14: 1-6.
